# Development and Psychometric Properties of the Sussex-Oxford Compassion Scales (SOCS)

**DOI:** 10.1177/1073191119860911

**Published:** 2019-07-29

**Authors:** Jenny Gu, Ruth Baer, Kate Cavanagh, Willem Kuyken, Clara Strauss

**Affiliations:** 1 University of Sussex, Falmer, East Sussex, UK; 2 University of Kentucky, Lexington, KY, US; 3 University of Oxford, Headington, Oxford, UK; 4 Sussex Partnership NHS Foundation Trust, Hove, UK

**Keywords:** compassion, self-compassion, self-report, measure, questionnaire, SOCS-O, SOCS-S

## Abstract

Compassion has received increasing societal and scientific interest in recent years. The science of compassion requires a tool that can offer valid and reliable measurement of the construct to allow examination of its causes, correlates, and consequences. The current studies developed and examined the psychometric properties of new self-report measures of compassion for others and for the self, the 20-item Sussex-Oxford Compassion for Others Scale (SOCS-O) and 20-item Sussex-Oxford Compassion for the Self Scale (SOCS-S). These were based on the theoretically and empirically supported definition of compassion as comprising five dimensions: (a) recognizing suffering, (b) understanding the universality of suffering, (c) feeling for the person suffering, (d) tolerating uncomfortable feelings, and (e) motivation to act/acting to alleviate suffering. Findings support the five-factor structure for both the SOCS-O and SOCS-S. Scores on both scales showed adequate internal consistency, interpretability, floor/ceiling effects, and convergent and discriminant validity.

Compassion is considered to be an innate, evolved capacity (Darwin, 1871; de Waal, 2009; Gilbert, 2005) and has long been emphasized to be a core human virtue in major contemplative and religious traditions (Dahlsgaard, Peterson, & Seligman, 2005). Recently, there has been a surge in scientific interest in compassion and increased recognition of the importance of both compassion for others and compassion for the self across multiple sectors of society, including health care, education, and the justice system (e.g., American Medical Association, 2001; Compassion in Education Foundation, 2016; Department of Health, 2013; Norko, 2005). Compassion is associated with a range of adaptive and prosocial characteristics and outcomes, such as greater well-being (Davidson & Schuyler, 2015), happiness (Mongrain, Chin, & Shapira, 2011), and reduced depressive symptoms (López, Sanderman, Ranchor, & Schroevers, 2018), and there is growing evidence that greater compassion can be cultivated through compassion-based interventions (CBIs; Kirby, Tellegen, & Steindl, 2017). The science of compassion requires a tool that can offer valid and reliable measurement of the construct to allow examination of its causes, correlates, and consequences (Strauss et al., 2016). This article reports on the development and psychometric properties of parallel measures of compassion for others and compassion for the self.

While there are many definitions of compassion, there has been a lack of consensus on its key defining features. The Oxford English Dictionary defines compassion solely in terms of an emotional response to suffering (“sympathetic pity and concern for the sufferings or misfortunes”). In psychological literature, compassion has been conceptualized as comprising recognition and awareness of suffering, emotionally connecting with that suffering, and the desire to act or acting to ease the suffering (Jazaieri et al., 2013; Kanov et al., 2004). Other definitions also highlight the integral role of distress tolerance, the ability to stay with difficult emotions when faced with suffering (Dalai Lama, 2002; Gilbert, 2010; Wispe, 1991), and common humanity, understanding that suffering is a universal human experience (Feldman & Kuyken, 2011; Neff, 2003; Pommier, 2010).

In the context of increasing and widespread interest in compassion and how it can be cultivated, there is a need to consolidate the range of theoretical conceptualizations of compassion into one comprehensive, operational definition. A recent position paper reviewed and consolidated a range of conceptualizations of compassion into one multifaceted, operational definition in an attempt to provide the clarity necessary to advance compassion research (Strauss et al., 2016). The definition conceptualizes compassion as a cognitive, affective, and behavioral process consisting of the following five elements: (a) recognizing suffering; (b) understanding the universality of suffering in human experience; (c) feeling for the person suffering and emotionally connecting with their distress; (d) tolerating any uncomfortable feelings aroused in response to the suffering (e.g., fear, disgust, distress) so that we remain accepting of and open to the person suffering; and (e) acting or being motivated to act to alleviate the suffering. As well as encompassing these elements, a key feature of compassion that distinguishes it from related states (e.g., empathy, kindness, sympathy) is that it arises specifically in response to suffering (Strauss et al., 2016). Consistent with theory that the process of compassion is broadly the same whether it is directed at the self or at others (Feldman & Kuyken, 2011; Gilbert, 2009, 2014), this five-element definition applies to both. That is to say recognizing suffering, and its universality, being able to tolerate elicited feelings, and acting to alleviate suffering can be directed equally to the self or others.

As well as being grounded in theory, this five-element conceptualization of compassion has also received empirical support. Gu, Cavanagh, Baer, and Strauss (2017) conducted a series of factor analytic studies to empirically examine the underlying conceptual structure of compassion using items from existing self-report compassion measures. Their findings showed support for a five-factor hierarchical structure of compassion consistent with Strauss et al.’s (2016) definition, with the five elements captured under an overarching compassion factor.

Although factor analyses of existing items support the five-element definition of compassion, existing measures of compassion fail to capture the breadth of all five elements. In addition to their review of conceptualizations of compassion, Strauss et al. (2016) also systematically reviewed nine existing questionnaire measures of self- and other-compassion^1^ and found that none of the scales comprehensively captured the construct of compassion, with some items from measures worded in conflict with the response scale, containing the word “compassion” and relying on respondents to define this term, appearing to tap into related constructs such as empathy and kindness, and not being related to suffering and thus arguably not capturing compassion. Many existing measures also have poor or inadequately tested psychometric properties, namely poor internal consistency and insufficient evidence for factor structure, with none of the reviewed measures scoring over 50% on the quality rating tool.

Given the lack of valid and reliable measures which comprehensively capture compassion, there is a need to develop new robust measures of compassion for the self and others in order to progress scientific investigation. Continued use of measures which are limited both in whether they fully capture the nature of compassion and their psychometric properties could lead to erroneous research findings which would be counterproductive for compassion research and practice. Key areas of research and clinical practice which would benefit from new robust measures of compassion include evaluating the causes and consequences of compassion and examining whether psychological interventions developed to explicitly or implicitly enhance people’s capacity for compassion for themselves and other people (e.g., Mindfulness-Based Stress Reduction: Kabat-Zinn, 1982; Mindfulness-Based Cognitive Therapy: Segal, Williams, & Teasdale, 2002, 2013; Compassion Focused Therapy: Gilbert, 2014; Mindful Self-Compassion: Neff & Germer, 2013) work through their hypothesized mechanism of action (i.e., improved compassion).

## The Current Program of Research

The current program of research aimed to address the lack of robust compassion measures by developing and psychometrically evaluating two parallel self-report measures of compassion based on Strauss et al.’s (2016) theoretically and empirically supported five-element definition of compassion: the Sussex-Oxford Compassion for Others Scale (SOCS-O) and the Sussex-Oxford Compassion for the Self Scale (SOCS-S). Self and other versions of the scale were developed in parallel in keeping with the theoretical literature on compassion which does not distinguish between the two (e.g., Feldman & Kuyken, 2011; Gilbert, 2009, 2014; Strauss et al., 2016). Developing compassion for self and other scales in parallel has the potential to empirically test this theory and to enhance understanding of the nature of the relationship between compassion for the self and compassion for others (Gu et al., 2017). Parallel scales will clarify the facets underlying compassion for self and others (theory would predict that the factor structure of both scales will mirror each other) and will also enable empirical examination of the overlap between the experience of compassion for self and others.

Development and validation of the SOCS-O and SOCS-S comprised four stages: (a) item generation and review through consultation with both experts and nonexperts, (b) item reduction using data from a sample of health care staff, (c) validation of the factor structure of measures and evaluation of their psychometric properties in a sample of health care staff, and (d) cross-validation of their factor structure and evaluation of their psychometric properties in a sample of University students. Health care staff were recruited in Stages 2 and 3 for a number of reasons. First, they represent a well-defined sample for whom compassion for self and others may be particularly salient on a daily basis, given their experience of providing care to others while working in an emotionally demanding profession. Second, in response to increasing research and societal interest in compassion in health care contexts; there has been a particular emphasis on creating a culture of compassion in the health care sector (e.g., American Medical Association, 2001; Department of Health, 2013; NHS England, 2017). This is linked to research indicating improvements in patient outcomes associated with increased compassionate care (e.g., Epstein et al., 2005; Sanghavi, 2006), acknowledgement of self-care as an integral part of providing effective care to others (NHS England, 2017), and reports of diminishing compassion for self and others in cases of work-related burnout (Joinson, 1992). Last, recruiting health care staff allowed for empirical testing of key research questions in this sample, including whether compassion for the self is related to providing compassionate care to others and whether enhanced compassion is linked to reduced work-related burnout. These questions were addressed in Stage 3 of this program of research.

The four stages followed best practice guidelines for measure development in terms of generating items in relation to a theoretically informed, operational definition and in consultation both with experts in the topic and nonexperts from a population likely to complete the measures in future research, assessing the content validity of items, reducing item pools to remove redundant items and create scales of manageable length, validating factor structures in independent samples to confirm a prespecified model for the measures, and assessing other psychometric properties, such as internal consistency and convergent and discriminant validity (e.g., Byrne, 2005; Costello & Osborne, 2005; Furr, 2011; Hinkin, 1998). All four stages received ethical approval from the Sciences & Technology Cross-Schools Research Ethics Committee of the University of Sussex. The method and results for each stage are presented in turn.

## Stage 1: Item Generation and Review

The aim of this stage was to generate and review items through consultation with both experts and nonexperts. To maximize content validity, we used the five-element definition of compassion to formulate items that closely related to each dimension. Items relating to self-compassion and other-compassion were generated in parallel. Items were generated and revised in consultation with experts in contemplative approaches purposively sampled to represent different cultural contexts across the globe and reviewed by nonexperts representative of the populations likely to complete the measure.

### Item Generation

#### Method

##### Participants and procedure

A total of 22 English-speaking experts in contemplative approaches (72.7% female; *M*_age_ = 43.50 years, *SD*_age_ = 11.62), defined as researchers and/or teachers in the fields of mindfulness and compassion with personal experience of contemplative practice (i.e., experience of cultivating mindfulness and/or compassion through contemplative meditation practices), were consulted to generate compassion items under the five elements identified by Strauss et al. (2016) and Gu et al. (2017). Participation was voluntary. Experts responded to e-mail invitations distributed through contemplative research and teacher networks. Experts had on average 10.86 years of personal contemplative practice experience (*SD* = 7.39). There were at least two experts from each of the six continents (Europe, Asia, Africa, North America, South America, Australia) and within each continental group, there was at least one representative from each expert group (researcher or teacher).

Interviews with experts were conducted by the first author over telephone or Skype. At least 24 hours prior to the interviews, experts were provided with an information sheet detailing the five elements of compassion, the interview procedure, scale instructions, preferred item characteristics (e.g., chosen response scale, response period, items worded as statements, both positively phrased and negatively phrased items), and good practice guidelines for formulating items (e.g., avoiding double-barreled items, keeping item wording concise, excluding frequency terms such as “often” and “sometimes”; DeVellis, 2016; Terwee et al., 2007). The intention was to develop measures which could be used widely, in many adult populations, and we therefore aimed to develop items which were succinct, clear, and understandable and in the scale instructions, defined the use of more ambiguous terms such as “suffering” (see the copy of the resulting scales in the supplementary materials available online, for scale instructions). The information sheet also informed experts of the intention to develop measures of both self- and other-compassion. Experts were asked to generate up to three parallel items that they thought best described each element of compassion for self and others.

#### Results

Altogether, experts generated 155 other-compassion items and 101 self-compassion items. All authors reviewed all generated items and came to a consensus regarding the set of items through the following iterative process. To retain as many generated items as possible, items were removed only if they were semantic duplicates and if they did not conceptually capture a particular element of compassion. Some items were also reworded to fit the response scale and parallel items were generated where these were lacking (e.g., generating an other-compassion version of an item which had only a self-compassion form). All universality of suffering items could be applied to both the self and others (e.g., “I understand that everyone experiences suffering at some point in their lives”) and were included in both self- and other-compassion item pools. The authors also compared all generated items with existing compassion measures included in Strauss et al.’s (2016) review to ensure good coverage of generated items; no items were added, removed, or changed based on this comparison. Following the iterative review by authors, the pool of items was reduced to 60 compassion for others items and 60 compassion for the self items.

### Item Review

#### Method and Results

Fifteen of the experts in contemplative approaches who contributed to the generation of the initial pool of items and 15 nonexperts (60.0% female; *M*_age_ = 28.27 years, *SD*_age_ = 5.08) reviewed the generated items. Nonexperts were undergraduate students at a University in the South of England with no prior experience of mindfulness meditation or who have not undertaken a contemplative or compassion-based course.

An anonymous online survey on Bristol Online Surveys (www.onlinesurveys.ac.uk) containing the 60 other-compassion and 60 self-compassion items, displayed under their relevant element, was administered to participants. The survey for experts asked them to consider whether each item adequately represents its relevant element and respond accordingly by selecting “yes” or “no.” The survey for nonexperts asked them to consider whether the wording of each item is clear and understandable (“yes” or “no”). It was agreed a priori that an item would be removed if more than 50% of experts responded “no,” indicating that it does not adequately represent its relevant element, or if more than 50% of nonexperts responded “no,” indicating that it is not clearly worded.

None of the items were removed based on the review by nonexperts. Three other-compassion items and two self-compassion items were deemed by experts to not adequately represent their relevant elements. These five items were removed, leaving 57 other-compassion items and 58 self-compassion items for Stage 2.

## Stage 2: Item Reduction

Stage 2 aimed to reduce the pool of self- and other-compassion items generated in Stage 1. To do this, we applied the theoretically and empirically supported five-factor model separately on the pool of self- and other-compassion items using confirmatory factor analysis (CFA) and selected items with the highest loadings on each factor.

### Method

#### Participants and Procedure

Participants were 1,017 health care staff working in a role that involves at least 1 day a week of direct contact with patients. Staff were recruited from public health care organizations in the South of the United Kingdom. Participation was voluntary. Of the 1,017 participants, 859 completed demographic questions, with the exception of age, which was completed by 843 participants. The mean age of the sample was 42.37 years (*SD* = 11.99; range: 18-77 years) and 79.6% were female (*n* = 684). Most of the sample were White (90.2%) and married, in a civil partnership, cohabiting, or in a long-term relationship (73.0%). In terms of level of education, 9 (1.0%) had no formal qualifications, 80 (9.3%) had some General Certificate of Secondary Education (GCSE; U.K. school qualifications received at age 16) or equivalent qualifications, 145 (16.9%) had some A Levels (U.K. school qualifications received at age 18) or equivalent qualifications, 391 (45.5%) had a bachelor’s degree or equivalent, and 234 (27.2%) had a higher degree, such as a master’s or doctoral degree. The majority of staff worked in nursing (30.2%), followed by allied health (18.5%), and ambulance services (10.4%); each remaining job role category comprised less than 10% of the sample. Participants completed an anonymous online survey on Qualtrics (www.qualtrics.com) containing several self-report measures.

#### Measures

##### Compassion items

This consisted of the 57 other-compassion items and 58 self-compassion items derived from Stage 1. The self- and other-compassion items appeared separately and their order was counterbalanced, such that for around half of participants, other- or self-compassion scales appeared first. Items were arranged such that they alternated among the five elements. Participants were instructed to indicate how true each statement was of them using a 5-point Likert-type scale ranging from 1 (*not at all true*) to 5 (*always true*).

Along with the compassion items, other measures of mindfulness, compassion, empathy, well-being, depression, anxiety, and stress were administered in the survey as part of a larger study and data on these are not reported on here.

#### Planned Data Analysis

Two five-factor CFA models, with items loading on respective factors from the five-element conceptualization of compassion (Strauss et al., 2016), were applied; one to the pool of other-compassion items and one to the pool of self-compassion items. Models used maximum-likelihood estimation with robust standard errors conducted in M*plus* version 7.4 (Muthén & Muthén, 1998-2015). As the aim of this stage was to select items for the resulting scales based on their standardized loadings on factors, model-data fit indices were not reported for this stage. Examining model-data fit alongside item reduction may bias which items are selected and a stronger test would be to validate the factor structures of the resulting scales in independent samples (Stages 3 and 4; Levine, 2005; Matsunaga, 2010). To create scales of manageable length for use in a variety of contexts, the four highest loading items were selected for each factor, creating 20-item self- and other-compassion measures.

### Results

#### Compassion for Others

Indeed, 932 staff completed other-compassion items and were included in the item selection for this scale. There were no missing item-level data; all 932 staff completed all other-compassion items. Table S1 (see supplementary materials available online) shows the standardized loadings of items on respective factors. The four highest loading items for each factor were selected for the SOCS-O and these are preceded by an asterisk. All standardized loadings were significant (*p* < .001) and all selected items had loadings greater than .40.

#### Compassion for the Self

A total of 947 participants completed self-compassion items and were included in the item selection for this scale. There were no missing item-level data; all 947 staff completed all self-compassion items. Table S2 (see supplementary materials available online) presents the standardized item loadings on respective factors. The four highest loading items for each factor were retained for the SOCS-S; these are preceded by an asterisk. All standardized loadings were significant (*p* < .001) and all selected items had loadings greater than .40.

## Stage 3: Validating Factor Structures Using CFA

Stage 3 applied CFA to data from a large, independent sample of health care staff to confirm the factor structures of the SOCS-O and SOCS-S. We hypothesized that the theoretically derived five-element model of compassion, which conceptualizes a hierarchical structure, whereby the five related components (recognizing, universality, feeling, tolerating, and acting) are elements of an overarching compassion factor, would be a good fit to data for both the SOCS-O and SOCS-S. CFA is the recommended approach for confirming, and testing hypotheses about, a preconceived factor structure (Costello & Osborne, 2005).

This stage also tested other psychometric properties of these scales, namely convergent and discriminant validity (the degree to which scales were related to other measures in ways consistent with theoretically derived hypotheses), interpretability (the extent to which qualitative meaning can be attached to quantitative scores; tested by comparing scale scores in subgroups of participants), internal consistency of total scale and subscale items (the extent to which items in a scale or subscale are correlated), and floor and ceiling effects (the percentage of respondents achieving the highest and lowest possible scores on scales).

For the criterion of interpretability to be met, Terwee et al. (2007) requires scale scores to be compared in at least four subgroups of participants. We examined whether SOCS-O and SOCS-S scores differed in relation to gender, length of previous meditation experience (four subgroups: no previous experience, less than a year, 1 to 5 years, over 5 years), level of education (five subgroups: no formal qualifications, GCSE/equivalent, A-level/equivalent, degree/equivalent, and higher degree/equivalent), and marital status (four subgroups: single, married/civil partnership/cohabiting/long-term relationship, separated/divorced, widowed). We predicted that there would be a significant gender difference in SOCS-O scores only, with females scoring higher than males, consistent with previous research showing that females score significantly higher on measures of compassion for others than males, but no gender difference for self-compassion (e.g., Neff & Pommier, 2013; Pommier, 2010). Based on previous research demonstrating that meditators reported significantly higher levels of both other-compassion and self-compassion compared with nonmeditator samples (e.g., Neff & Pommier, 2013), we also hypothesized that length of previous meditation experience would have a significant effect on SOCS-O and SOCS-S scores, with subgroups with more meditation experience scoring significantly higher on these scales compared with those with less meditation experience. Due to lack of research and compelling reasons to expect differences in scale scores in relation to level of education and marital status, we did not make any predictions for these variables, but explored their findings.

For the criterion of convergent and discriminant validity to be met, Terwee et al. (2007) requires prespecified hypotheses to be made, at least three quarters of results to be consistent with hypotheses, and in relation to convergent validity, at least two of the correlations to be large (*r* ≥ .50; Barker, Pistrang, & Elliott, 2002). Theoretically derived hypotheses for this criterion are given in the planned data analysis subsection after describing the measures used to test this property. We explored the internal consistency of total scale and subscale items on the SOCS-O and SOCS-S and floor and ceiling effects of these scales using analyses described in the planned data analysis subsection.

### Method

#### Participants and Procedure

An independent sample of 1,319 health care staff completed an anonymous online survey on Qualtrics containing self-report measures. Staff were recruited from public health care organizations in the south of the United Kingdom. Participation was voluntary. Indeed, 1,132 to 1,137 participants completed demographic questions, with the exception of age, which was completed by 1,123 participants. The mean age of the sample was 44.83 years (*SD* = 11.30; range: 18-74 years) and 83.1% were female (*n* = 945). Most of the sample were White (89.7%) and married, in a civil partnership, cohabiting, or in a long-term relationship (76.7%). In terms of level of education, 12 (1.1%) had no formal qualifications, 144 (12.7%) had some GCSEs (U.K. school qualifications received at age 16) or equivalent qualifications, 201 (17.8%) had some A Levels (U.K. school qualifications received at age 18) or equivalent qualifications, 502 (44.3%) had a bachelor’s degree or equivalent, and 273 (24.1%) had a higher degree, such as a master’s or doctoral degree. The majority of staff worked in nursing (39.2%), followed by allied health services (15.2%) and administrative and clerical roles (15.3%); remaining job role categories comprised less than 10% of the sample.

#### Measures

With the exception of the SOCS-O and SOCS-S, the below measures were selected because they are theoretically expected to be related in particular ways to self- and/or other-compassion.

##### Sussex-Oxford Compassion for Others Scale and Sussex-Oxford Compassion for the Self Scale

The 20-item SOCS-O and 20-item SOCS-S derived from Stage 2 appeared separately, either at the start or the end of the survey, and their order was counterbalanced. For each scale, items were arranged such that they alternated among the five elements. Participants were instructed to indicate how true each statement was of them using a 5-point Likert-type scale ranging from 1 (*not at all true*) to 5 (*always true*). A copy of the SOCS-O and SOCS-O, with scoring information, is included in the supplementary materials, which is available online.

##### Five-Facet Mindfulness Questionnaire (FFMQ) 15-item version (Baer, Carmody, & Hunsinger, 2012)

The 15-item FFMQ (FFMQ-15) is a short form of the 39-item FFMQ (FFMQ-39) and measures the general tendency to be mindful in everyday life. It includes the same five facets as the long form: observing, describing, acting with awareness, nonjudging of inner experience, and nonreactivity to inner experience. The factor structure of the FFMQ-15 is consistent with that of the FFMQ-39, there are large correlations between total facet scores of the short and long forms, and the two FFMQ versions do not differ significantly from each other in terms of convergent validity (Gu et al., 2016). Previous research (Baer, Smith, Hopkins, Krietemeyer, & Toney, 2006; Gu et al., 2016; Williams, Dalgleish, Karl, & Kuyken, 2014) found that in nonmeditator samples, a four-factor hierarchical structure without the “observing” facet provided a superior fit compared with a five-factor hierarchical structure. As it is likely that our current sample has little or no previous meditation experience, “observing” items were excluded. FFMQ-15 items were rated on a 5-point Likert-type scale ranging from 1 (*never or very rarely true*) to 5 (*very often or always true*). Cronbach’s alpha for FFMQ-15 items (excluding observing items) was .80.

##### Self-Compassion Scale–Short form (SCS-12; Raes, Pommier, Neff, & Van Gucht, 2011)

This 12-item measure is a short form of the original 26-item scale (Neff, 2003). The SCS-12 was found to have the same factor structure as the long form, with six factors loading on a higher order self-compassion factor: self-kindness, self-judgment, common humanity, isolation, mindfulness, and overidentification (Raes et al., 2011). Items were rated on a 5-point Likert-type scale ranging from 1 (*almost never*) to 5 (*almost always*). Cronbach’s alpha for SCS-12 items was .88.

##### Santa Clara Brief Compassion Scale (SCBCS; Hwang, Plante, & Lackey, 2008)

The 5-item SCBCS is a short form of the 21-item Compassionate Love Scale (Sprecher & Fehr, 2005) and measures compassion toward strangers and humankind at large. Responses to items were given on a 7-point Likert-type scale ranging from 1 (*not at all true of me*) to 7 (*very true of me*). Of all the existing other-compassion measures reviewed by Strauss et al. (2016), the SCBCS was the shortest measure which obtained the highest quality rating. Cronbach’s alpha for SCBCS items was .91.

##### Interpersonal reactivity index (IRI; Davis, 1980)

The 28-item IRI is a multidimensional measure of dispositional empathy, with four subscales: perspective taking, fantasy, empathic concern, and personal distress. Responses were given on a 5-point Likert-type scale from 1 (*does not describe me well*) to 5 (*describes me very well*). Following previous research (e.g., Neff & Pommier, 2013; Pommier, 2010), we excluded the fantasy subscale, because it is not regarded as assessing a core part of empathy. Cronbach’s alphas were .79 (perspective taking), .75 (empathic concern), and .76 (personal distress).

##### Depression, Anxiety, and Stress Scale–Short form (DASS; Henry & Crawford, 2005)

The 21-item shortened version of the DASS measures the severity of core symptoms associated with depression, anxiety, and stress. Participants were asked to indicate the presence of each symptom over the past week. Responses were given on a 4-point Likert-type scale ranging from 0 (*never*) to 3 (*almost always*). Cronbach’s alphas were .92 (depression), .81 (anxiety), and .86 (stress).

##### Short Warwick-Edinburgh Mental Well-Being Scale (SWEMWBS; Stewart-Brown et al., 2009)

The seven-item SWEMWBS is a measure of positive mental well-being. This measure involves rating items on a 5-point Likert scale ranging from 1 (*none of the time*) to 5 (*all of the time*). Participants were asked to rate items based on their experience over the past 2 weeks. Cronbach’s alpha for SWEMWBS items was .89.

##### Maslach Burnout Inventory–Human Services Survey (MBI-HSS; Maslach, Jackson, & Leiter, 1981)

The 22-item MBI-HSS was designed to measure work-related burnout in professionals working in the human services such as health care and consists of three distinct subscales: emotional exhaustion, depersonalization, and personal accomplishment. Participants were asked about the frequency with which they have certain experiences and items were answered on a 7-point Likert-type scale ranging from 0 (*never*) to 6 (*every day*). The MBI-HSS was administered to a subset of participants in this sample (*n* = 115).^2^ Cronbach’s alphas were .90 (emotional exhaustion), .75 (depersonalization), and .78 (personal accomplishment).

#### Planned Data Analysis

Three CFA models were tested for the 20-item SOCS-O and 20-item SOCS-S: (a) a one-factor model in which all items are direct indicators of a single compassion factor; (b) a five-factor correlated model, with items loading on respective factors from the five-element definition of compassion (Strauss et al., 2016); and (c) a five-factor hierarchical model, where the five factors load on an overarching compassion factor. All CFA models used maximum likelihood estimation with robust standard errors conducted in M*plus* version 7.4 (Muthén & Muthén, 1998-2015).

The following five fit indices were used to indicate model-data fit: the comparative fit index (CFI; Bentler, 1990), root mean square error of approximation (RMSEA; Steiger, 1990), nonnormed fit index (NNFI; Bentler & Bonett, 1980), standardized root mean square residual (SRMR), and Akaike information criterion (AIC; Akaike, 1974). Rules of thumb cutoff criteria for determining adequate fit using these indices can be arbitrary and affected by numerous factors such as sample size, data distribution, and model complexity and specifications (e.g., Chen, Curran, Bollen, Kirby, & Paxton, 2008; Marsh, Hau, & Wen, 2004), such that a model may fit the data even when one or more indices suggest inadequate fit (Schermelleh-Engel, Moosbrugger, & Müller, 2003). Consequently, researchers do not recommend their use as absolute, universally applied rules for assessing fit (e.g., Hu & Bentler, 1999; Marsh et al., 2004).

Given these considerations, following Williams et al. (2014), we used both liberal and conservative cutoff points for acceptable fit for the CFI, RMSEA, NNFI, and SRMR: the CFI and NNFI should be close to or greater than .90 (liberal) or .95 (conservative), RMSEA should be .10 or less (liberal) or .06 or less (conservative), and SRMR should be less than .10 (liberal) or .05 (conservative). We also considered the significance of factor intercorrelations and loadings when interpreting model fit. The AIC was used to compare the fit of the models, with lower values indicating superior fit. Although the chi-square test of model fit was reported, the significance of this statistic was not used to indicate model fit because of its hypersensitivity (e.g., to nonnormality, large sample sizes, large correlations between variables; R, B. Kline, 2015).

Internal consistency of total scale and subscale items on the SOCS-O and SOCS-S was assessed using both Cronbach’s alpha and omega total coefficients; values greater than or equal to .70 indicate good internal consistency (Terwee et al., 2007), although for psychological constructs, values below .70 are acceptable (P. Kline, 1999). Cronbach’s alphas were computed using SPSS version 24 (IBM, 2016) and omega total estimates were computed using McNeish’s (2017) Excel spreadsheet using standardized item loadings from the CFA model with superior fit. Omega total estimates were calculated alongside Cronbach’s alpha given well-documented methodological limitations of the latter, such as overly rigid assumptions which are commonly violated and poor performance when compared with alternative measures such as omega total (e.g., McNeish, 2017).

Floor and ceiling effects of the SOCS-O and SOCS-S were examined by calculating the percentage of respondents achieving the highest and lowest possible scores; less than 15% of the sample should receive the highest or lowest score (Terwee et al., 2007). Interpretability was tested by conducting independent *t* tests and one-way analyses of variance, and reporting means, standard deviations, and effect sizes, to examine whether total scale scores differ in relation to: gender, length of previous meditation experience, level of education, and marital status.

Convergent and discriminant validity were tested by examining whether each scale correlated with the measures detailed in the measures subsection in line with the below predictions. We predicted that the SOCS-O and SCBCS, both scales measuring compassion for others, would be significantly correlated at *r* ≥ .50. Similarly, the SOCS-S and SCS-12, both measures of self-compassion, were expected to be significantly correlated at *r* ≥ .50. We expected the SOCS-O to be significantly correlated with the empathic concern and perspective taking subscales of the IRI at *r* ≥ .50. However, although we would expect the SOCS-O to be significantly and negatively related to the personal distress subscale of the IRI, a prediction was not made as to the size of this relationship, because unlike the other two subscales, almost all personal distress items are worded ambiguously in terms of target and can be interpreted in relation to the self rather than others (e.g., “Being in a tense emotional situation scares me” and “I sometimes feel helpless when I am in the middle of a very emotional situation”). Previous research has found just a small-moderate, negative correlation between compassion for others and the personal distress subscale of the IRI (Pommier, 2010). Findings supporting these predictions would provide evidence for convergent validity.

Consistent with research which found significant correlations, ranging in size from small-moderate to large, between self-compassion and mindfulness, positive mental health, and well-being (e.g., Durkin, Beaumont, Martin, & Carson, 2016; López et al., 2018; Neff, 2003; Pommier, 2010), but no such relationships between compassion for others and these constructs (e.g., Durkin et al., 2016; López et al., 2018; Pommier, 2010), we predicted that there would be significant correlations between the SOCS-S and the FFMQ-15, SWEMWBS, and all subscales of the DASS small-moderate in size (positive for the FFMQ-15 and SWEMWBS and negative for DASS subscales). Findings supporting these predictions would provide evidence for convergent and discriminant validity for the SOCS-S. It is possible that the lack of significant correlations between compassion for others and mindfulness, well-being, and mental health was due to limitations of existing compassion measures (Strauss et al., 2016) and we therefore explored these findings but did not make specific predictions about the relationships between the SOCS-O and these constructs. Similarly, research has found a moderate-large, negative, significant correlation between self-compassion and burnout but no such relationship between compassion for others and burnout (e.g., Durkin et al., 2016). We therefore expected significant, moderate-large correlations between the SOCS-S and subscales of the MBI-HSS (negative for emotional exhaustion and depersonalization and positive for personal accomplishment) but did not make predictions for the SOCS-O and MBI-HSS.

Moreover, self- and other-compassion are theoretically overlapping constructs and the process of compassion is the same whether it is directed at the self or at others. However, research into the relationship between self- and other-compassion has found no more than a small relationship between these constructs (Durkin et al., 2016; López et al., 2018; Neff & Pommier, 2013; Pommier, 2010). It is currently unclear whether the little or no empirical overlap between self- and other-compassion is due to limitations of the measures used in these studies (e.g., Strauss et al., 2016; Williams et al., 2014) or indicates that these two forms of compassion are largely distinct. Thus, no specific hypotheses were made regarding the correlation between the SOCS-O and SOCS-S, but these findings were explored. Last, to test discriminant validity, none of the relationships between the SOCS-O or SOCS-S and other measures were expected to correlate so highly (*r* ≥ .80; Field, 2013) as to indicate that they were the same construct (e.g., compassion and empathy) or that measures were indistinguishable (e.g., SOCS-O/SOCS-S and existing compassion scales).

### Results

#### Confirmatory Factor Analysis

##### Compassion for others

However, 1,242 health care staff completed the SOCS-O and were included in the CFA. There were no missing item-level data; all 1,242 participants completed all SOCS-O items. Table 1 shows the fit indices for the three CFA models. Almost all fit indices indicated poor fit of the one-factor model to the data, suggesting that items are not direct indicators of an overarching compassion factor. All fit indices indicated good fit of the five-factor and five-factor hierarchical models according to both liberal and conservative criteria. All loadings of items on factors in these two models were significant. All factor intercorrelations in the five-factor model were significant. In the five-factor hierarchical model, all loadings of factors on the overarching compassion factor were significant, suggesting that the five factors are elements of an overall compassion for others construct. Based on both the fit indices and significance of factor loadings, the five-factor hierarchical model can be interpreted as best fitting the data. Table S3 (see supplementary materials available online) presents the standardized loadings of items on to factors in the five-factor hierarchical model for the SOCS-O and Table S4 (see supplementary materials available online) the standardized factor loadings in the five-factor hierarchical model. Table S5 (see supplementary materials available online) shows the correlations between total scale and subscale scores on the SOCS-O in the health care staff validation sample.

**Table 1.** Fit Indices for Compassion Models Tested in Both Validation Samples (Stages 3 and 4).

Scale	Sample	Model	CFI	RMSEA [90% CI]	NNFI	SRMR	χ^2^ (*df*)	AIC
Compassion for Others	1,242 Health care staff (Stage 3)	One-factor	.718	.122 [.119, .126]	.685	**.089**	3338.294 (170)	42176.726
		Five-factor	**.973**	**.039 [.035, .043]**	**.968**	**.028**	466.435 (160)	38170.026
		Five-factor hierarchical^a^	**.972**	**.039 [.035, .043]**	**.968**	**.029**	475.491 (165)	38174.744
	371 Students (Stage 4)	One-factor	.632	.126 [.119, .132]	.589	.107	1163.712 (170)	14200.646
		Five-factor	**.966**	**.040 [.030, .049]**	**.959**	**.045**	252.665 (160)	13104.222
		Five-factor hierarchical^a^	**.964**	**.040 [.030, .049]**	**.959**	**.047**	261.210 (165)	13103.945
Compassion for the Self	1,216 Health care staff (Stage 3)	One-factor	.638	.142 [.139, .146]	.596	.132	4360.676 (170)	51699.527
		Five-factor	**.947**	**.056 [.052, .060]**	**.937**	**.050**	775.599 (160)	46658.552
		Five-factor hierarchical^a^	**.939**	**.059 [.056, .063]**	**.930**	**.068**	871.920 (165)	46772.251
	371 Students (Stage 4)	One-factor	.580	.156 [.149, .163]	.530	.155	1703.097 (170)	16986.098
		Five-factor	**.930**	**.065 [.058, .073]**	**.917**	**.069**	413.800 (160)	15362.973
		Five-factor hierarchical^a^	**.925**	**.067 [.059, .074]**	**.914**	**.084**	437.055 (165)	15380.924

*Note*. AIC = Akaike information criterion; CFI = comparative fit index; CI = confidence interval; NNFI = nonnormed fit index; RMSEA = root mean square error of approximation; SRMR = standardized root mean square residual. Bold indices (CFI, RMSEA, NNFI, and SRMR) indicate acceptable fit according to liberal cutoff criteria when rounded up or down to two decimal places.

a Five-factor hierarchical refers to a model in which all five factors load on an overarching compassion factor.

##### Compassion for the self

A total of 1,216 health care staff completed the SOCS-S and were included in the CFA. There were no missing item-level data; all 1,216 participants completed all SOCS-S items. Table 1 presents the fit indices for the three CFA models. All indices suggested poor fit of the one-factor model but adequate fit of the five-factor and five-factor hierarchical models. All item loadings in the two five-factor models were significant. All factor intercorrelations in the five-factor model were significant and all factor loadings in the five-factor hierarchical model were significant, suggesting that the five factors are related and are elements of an overall compassion for the self-construct. Based on both the fit indices and significance of factor loadings, the five-factor hierarchical model can be seen as best fitting the data. Table S6 (see supplementary materials available online) displays the standardized item loadings in the five-factor hierarchical model for the SOCS-S and Table S4 (see supplementary materials available online) the standardized factor loadings in the five-factor hierarchical model. Table S5 (see supplementary materials available online) presents the correlations between total scale and subscale scores on the SOCS-S in the staff validation sample.

##### Internal consistency

Omega total estimates, calculated using standardized item loadings from five-factor hierarchical models, ranged from .76 to .97 for total SOCS-O scale and subscale items and from .74 to .97 for total SOCS-S scale and subscale items (Table 2). Cronbach’s alphas ranged from .74 to .94 for total SOCS-O scale and subscale items and from .75 to .93 for total SOCS-S scale and subscale items (Table 2). These values are considered adequate for measures of psychological constructs (P. Kline, 1999; Terwee et al., 2007).

**Table 2.** Cronbach’s Alpha and Omega Total Coefficients for SOCS-O and SOCS-S Scale and Subscale Items in Both Validation Samples (Stages 3 and 4).

	Compassion for others				Compassion for the self			
	1,319 Health care staff (Stage 3)		371 Students (Stage 4)		1,319 Health care staff (Stage 3)		371 Students (Stage 4)	
	Alpha	Omega	Alpha	Omega	Alpha	Omega	Alpha	Omega
Total scale	.94	.97	.90	.96	.93	.97	.91	.97
Recognising suffering	.89	.90	.86	.86	.88	.88	.85	.85
Understanding the universality of suffering	.92	.92	.89	.89	.92	.92	.91	.91
Feeling for the person suffering	.80	.80	.73	.73	.84	.85	.84	.85
Tolerating uncomfortable feelings	.74	.76	.61	.65	.75	.74	.72	.73
Acting or being motivated to act to alleviate suffering	.91	.91	.86	.86	.91	.92	.90	.90

*Note.* SOCS-O = Sussex-Oxford Compassion for Others Scale; SOCS-S = Sussex-Oxford Compassion for the Self Scale.

#### Floor and Ceiling Effects

Less than 15% of the sample received the highest score (100) or lowest score (20) on the SOCS-O and SOCS-S; 0.1% and 0.2% of participants received the lowest possible score on the SOCS-O and SOCS-S, respectively, and 1.6% and 0.3% of participants received the highest possible score on the SOCS-O and SOCS-S, respectively, suggesting that both scales capture variability in responses.

#### Interpretability

Table 3 displays the means and standard deviations of total SOCS-O and SOCS-S scores across subgroups of participants. As predicted, females scored significantly higher on the SOCS-O compared with males, *t*(1118) = 5.97, *p* < .001, *d* = 0.47, but there was no significant difference between males and females in SOCS-S scores, *t*(1115) = 0.04, *p* = .965, *d* = 0.003. Length of previous meditation experience significantly affected scores on both the SOCS-O, *F*(3) = 5.53, *p* = .001, and SOCS-S, *F*(3) = 13.89, *p* < .001, as expected. Scores on both scales were significantly lower for those without any meditation experience, compared with those with 1 to 5 years’ experience, SOCS-O: *t*(1114) = 3.25, *p* = .001, *d* = 0.31; SOCS-S: *t*(1111) = 3.10, *p* = .002, *d* = 0.29, and over 5 years’ experience, *t*(1114) = 2.75, *p* = .006, *d* = 0.34; SOCS-S: *t*(1111) = 5.88, *p* < .001, *d* = 0.74. Additionally, those with over 5 years’ meditation experience scored significantly higher on the SOCS-S compared with both participants with less than a year’s experience, *t*(1111) = 4.81, *p* < .001, *d* = 0.79, and those with 1 to 5 years’ experience, *t*(1111) = 3.04, *p* = .002, *d* = 0.48. In terms of level of education, there was a significant difference in SOCS-S scores only, *F*(4) = 3.51, *p* = .007. The only significant contrast was between those with GCSEs (U.K. school qualifications received at age 16) or equivalent qualifications and those with higher degrees, *t*(1119) = 3.05, *p* = .002, *d* = 0.30. There was no significant effect of marital status on SOCS-O, *F*(3) = 1.02, *p* = .384, or SOCS-S scores, *F*(3) = 1.22, *p* = .300.

**Table 3.** Means and Standard Deviations of Total SOCS-O and SOCS-S Scores for all Participants and Participant Subgroups Using Available Data From Both Validation Samples (Stages 3 and 4).

	Total SOCS-O		Total SOCS-S	
	1,319 Health care staff (Stage 3)	371 Students (Stage 4)	1,319 Health care staff (Stage 3)	371 Students (Stage 4)
All participants	82.16 (9.73); *n* = 1,238	81.16 (8.56); *n* = 371	70.79 (11.65); *n* = 1,204	69.66 (11.11); *n* = 371
Gender				
Female	83.03 (9.28); *n =* 941	81.62 (8.35); *n* = 326	70.93 (11.40); *n* = 937	69.73 (11.12); *n* = 326
Male	78.42 (10.29); *n* = 179	78.12 (9.54); *n* = 42	70.97 (12.60); *n* = 180	69.48 (11.47); *n* = 42
Length of previous meditation experience				
No previous experience	81.58 (9.92); *n* = 800	80.51 (8.52); *n* = 283	70.05 (11.74); *n* = 798	68.57 (11.20); *n* = 283
Less than a year	82.66 (8.24); *n* = 109	83.47 (8.01); *n* = 49	69.95 (10.46); *n* = 107	72.08 (11.52); *n* = 49
1 to 5 Years	84.44 (8.43); *n* = 139	82.83 (9.18); *n* = 36	73.30 (10.33); *n* = 139	74.33 (8.20); *n* = 36
Over 5 years	84.86 (9.49); *n* = 70	85.00 (6.56); *n* = 3	78.35 (10.78); *n* = 71	77.00 (5.29); *n* = 3
Level of education				
No formal qualifications	77.83 (14.17); *n* = 12	—	64.67 (17.84); *n =* 12	—
GCSE or equivalent	80.84 (10.55); *n* = 143	—	68.40 (12.30); *n* = 141	—
A-level or equivalent	83.26 (9.75); *n* = 200	—	71.81 (11.57); *n* = 200	—
Degree (e.g., BA, BSc) or equivalent	82.18 (9.14); *n* = 500	—	70.91 (11.01); *n =* 500	—
Higher degree (e.g., MA, MSc, PhD) or equivalent	82.54 (9.54); *n* = 272	—	72.04 (11.61); *n* = 271	—
Marital status				
Single	81.01 (9.66); *n* = 152	81.27 (8.44); *n* = 315	69.85 (12.02); *n* = 154	69.30 (11.13); *n* = 315
Married/civil partnership/cohabiting/long-term relationship	82.37 (9.59); *n* = 868	80.53 (9.38); *n* = 55	71.33 (11.52); *n* = 864	71.53 (10.93); *n* = 55
Separated/divorced	82.86 (9.74); *n* = 96	—	69.71 (11.70); *n* = 95	—
Widowed	82.23 (10.91); *n* = 14	—	69.36 (8.28); *n =* 14	—

*Note.* SOCS-O = Sussex-Oxford Compassion for Others Scale; SOCS-S = Sussex-Oxford Compassion for the Self Scale; GCSE = General Certificate of Secondary Education. Standard deviations are given in parentheses.

#### Convergent and Discriminant Validity

Table 4 shows the correlation coefficients between total scale and subscale scores on the SOCS-O and SOCS-S and other constructs. Consistent with predictions, the SOCS-O had significant and large correlations with the SCBCS and empathic concern and perspective taking subscales of the IRI, and the SOCS-S had a significant and large correlation with the SCS-12. The SOCS-O was also significantly and negatively related to the IRI personal distress subscale. Additionally, the SOCS-S was significantly correlated in expected directions with the FFMQ-15, SWEMWBS, and DASS subscales, with correlations ranging from moderate-large to large in size. We also found significant, small-moderate correlations between the SOCS-O and the FFMQ-15 and SWEMWBS, and small, but significant, negative relationships between the SOCS-O and stress and depression (DASS). As predicted, the SOCS-S had significant and moderate-large correlations in expected directions with all subscales of the MBI-HSS. Although we did not make specific predictions for the SOCS-O, it was found to have significant, small-moderate correlations with MBI-HSS depersonalization (negative direction) and personal accomplishment (positive direction). Taken together, at least three quarters of results were consistent with predictions, at least two correlations were large (*r* ≥ .50), and none were *r* ≥ .80, providing support for the convergent and discriminant validity of total SOCS-O and SOCS-S scores in this health care staff sample. It should be noted, however, that there is a larger range of correlations between SOCS-O/SOCS-S subscales and the aforementioned measures.

**Table 4.** Correlation Coefficients Between Total Scores on the SOCS-O and SOCS-S and Other Constructs in Both Health Care Staff (Nonitalicized values) and Student (Values in Italics) Validation Samples (Stages 3 and 4).

	FFMQ-15^a^	SCBCS	SCS-12	IRI-EC	IRI-PD	IRI-PT	SWEMWBS	DASS-S	DASS-A	DASS-D	MBI-HSS EE^b^	MBI-HSS D^b^	MBI-HSS PA^b^
SOCS-O	.26***	.65***	.18***	.64***	−.16***	.54***	.24***	−.07*	−.03	−.07*	−.10	−.24*	.29**
	.19***	.59***	.14**	.59***	−.15**	.40***	.25***	−*.05*	−*.05*	−.18**			
Recognising suffering	.21***	.47***	.15***	.46***	−.18***	.40***	.22***	−.04	.01	−.05	−.07	−.16	.23*
	.11*	.36***	*.04*	.29***	−.17**	.22***	.14**	*.09*	*.09*	−*.03*			
Understanding the universality of suffering	.20***	.35***	.13***	.38***	−.08**	.35***	.19***	−.04	−.09**	−.06*	−.03	−.28**	.16
	.22***	.22***	.20***	.33***	−.10*	.23***	.30***	−.18***	−.19***	−.29***			
Feeling for the person suffering	.19***	.67***	.12***	.69***	−.07*	.50***	.17***	−.04	−.01	−.06	−.09	−.20*	.23*
	*.07*	.67***	*.04*	.65***	*.06*	.37***	.12*	*.06*	*.05*	−*.03*			
Tolerating uncomfortable feelings	.29***	.57***	.23***	.53***	−.24***	.52***	.27***	−.13***	−.08**	−.12***	−.20*	−.19*	.35***
	.24***	.38***	.18***	.41***	−.27***	.36***	.23***	−.18***	−.17**	−.22***			
Acting or motivation to act to alleviate suffering	.16***	.59***	.09**	.57***	−.13***	.44***	.16***	−.01	.04	−.01	−.04	−.16	.25**
	*.07*	.58***	*.04*	.52***	−*.09*	.35***	.12*	*.05*	*.03*	−*.10*			
SOCS-S	.55***	.23***	.65***	.23***	−.24***	.38***	.57***	−.43***	−.36***	−.45***	−.34***	−.36***	.31**
	.57***	.11*	.63***	.22***	−.29***	.29***	.54***	−.39***	−.39***	−.49***			
Recognising suffering	.31***	.22***	.30***	.23***	−.11***	.24***	.31***	−.20***	−.15***	−.18***	−.16	−.23*	.24**
	.21***	*.07*	*.07*	.19***	−*.04*	.12*	.24***	−*.08*	−.11*	−.19***			
Understanding the universality of suffering	.23***	.29***	.21***	.32***	−.10**	.34***	.25***	−.12***	−.19***	−.15***	−.11	−.22*	.17
	.20***	.14**	.22***	.34***	−*.09*	.27***	.26***	−.25***	−.17**	−.29***			
Feeling for the person suffering	.50***	.15***	.66***	.14***	−.19***	.31***	.53***	−.41***	−.32***	−.44***	−.27**	−.26**	.25* *
	.52***	.11*	.63***	.13**	−.26***	.23***	.48***	−.33***	−.34***	−.43***			
Tolerating uncomfortable feelings	.59***	.16***	.70***	.12***	−.35***	.31***	.59***	−.50***	−.41***	−.51***	−.40***	−.38***	.28**
	.63***	*.05*	.71***	*.04*	−.38***	.22***	.53***	−.46***	−.46***	−.46***			
Acting or motivation to act to alleviate suffering	.50***	.12***	.63***	.13***	−.19***	.27***	.54***	−.42***	−.32***	−.45***	−.35***	−.32**	.28**
	.51***	*.06*	.60***	.12*	−.27***	.20***	.44***	−.30***	−.32***	−.39***			

*Note*. DASS-A = Depression, Anxiety, and Stress Scale (anxiety subscale); DASS-D = Depression, Anxiety, and Stress Scale (depression subscale); DASS-S = Depression, Anxiety, and Stress Scale (stress subscale); FFMQ = Five Facet Mindfulness Questionnaire; IRI-EC = interpersonal reactivity index (empathic concern subscale); IRI-PD = interpersonal reactivity index (personal distress subscale); IRI-PT = interpersonal reactivity index (perspective taking subscale); MBI-HSS D = Maslach Burnout Inventory–Human Services Survey (depersonalization subscale); MBI-HSS EE = Maslach Burnout Inventory–Human Services Survey (emotional exhaustion subscale); MBI-HSS PA = Maslach Burnout Inventory–Human Services Survey (personal accomplishment subscale); SCBCS = Santa Clara Brief Compassion Scale; SCS-12 = 12-item Self-Compassion Scale; SOCS-O = Sussex-Oxford Compassion for Others Scale; SOCS-S = Sussex-Oxford Compassion for the Self Scale; SWEMWBS = Short Warwick–Edinburgh Mental Well-being Scale. Nonitalicized values are correlations from the sample of 1,319 health care staff (Stage 3). Values in italics are correlations from the sample of 371 students (Stage 4).

a Items from the observing subscale were excluded from the total FFMQ-15 score. ^b^The MBI-HSS was administered to a subset of the Stage 3 health care staff sample (*n* = 115). Students (Stage 4) did not complete this measure.

* *p* < .05. **p < .01. ***p < .001.

#### Relationship Between Compassion for the Self and Others

Health care staff scored significantly higher on the SOCS-O compared with the SOCS-S, *t*(1126) = 32.29, *p* < .001, *d* = 1.05, 95% confidence interval [0.98, 1.13]. Table S5 (see supplementary materials available online) shows the correlations between total scale and subscale scores on the SOCS-O and SOCS-S in the Stage 3 sample. Total scores were found to significantly correlate with a medium-large effect size at *r* = .40. Moreover, all SOCS-O and SOCS-S subscales were significantly correlated, with coefficients ranging between *r* = .15 (e.g., between the other-compassion acting subscale and self-compassion feeling subscale) and .78 (between other-compassion and self-compassion universality of suffering subscales). However, the correlation between total scale scores may be artificially inflated given that the wording of three of the four items from the universality of suffering subscale was the same for both scales. We therefore calculated the correlation between total SOCS-O and SOCS-S scores excluding the universality subscale and found these to be significantly and moderately correlated at *r* = .30 (*p* < .001).

## Stage 4: Cross-Validating Factor Structures Using CFA

Stage 4 applied CFA to data from a sample of university students to cross-validate the factor structures of the SOCS-O and SOCS-S. As in Stage 3, we hypothesized that the five-element model of compassion, which conceptualizes a hierarchical structure, would be a good fit to data for both the SOCS-O and SOCS-S.

### Method

#### Participants and Procedure

A sample of 371 undergraduate university students completed an anonymous online survey on Qualtrics containing self-report measures. Students were studying psychology in a university in the south of the United Kingdom. The mean age of the sample was 19.63 years (*SD* = 3.14; range: 18-45 years) and 87.9% were female (*n* = 326). Most of the sample were White (85.7%) and single (84.9%).

#### Measures

The measures used in Stage 3 were administered to students, with the exception of the MBI-HSS. Cronbach’s alphas for these measures were as follows: .80 (FFMQ-15 without observing items), .87 (SCS-12), .91 (SCBCS), .81 (IRI perspective taking), .78 (IRI empathic concern), .80 (IRI personal distress), .84 (DASS stress), .82 (DASS anxiety), .89 (DASS depression), and .86 (SWEMWBS). The SOCS-O and SOCS-S appeared separately, either at the start or end of the survey, and their order was counterbalanced.

#### Planned Data Analysis

Analyses in this stage mirrored those in Stage 3, with the exception of interpretability; in this student sample, data on level of education was not obtained. The same criteria and/or predictions were used to determine model fit and adequate psychometric properties.

### Results

#### Confirmatory Factor Analysis

##### Compassion for others

All 371 students completed all SOCS-O items and were included in the CFA. Table 1 displays the fit indices for the three CFA models tested on the SOCS-O in this sample. As with the CFA findings from Stage 3, fit indices indicated poor fit of the one-factor model but good fit of the five-factor and five-factor hierarchical models. All item loadings in these two models were significant. Factor intercorrelations in the five-factor model were significant and all loadings of factors on the overarching compassion factor in the five-factor hierarchical model were significant, indicating that the five factors are related and elements of an overall other-compassion construct. Based on both the fit indices and significance of factor loadings, the five-factor hierarchical model can be interpreted as best fitting the data. Tables S3 and S4 (see supplementary materials available online) show standardized SOCS-O item loadings and factor loadings, respectively, in the five-factor hierarchical model. Table S7 (see supplementary materials available online) shows correlations between total SOCS-O scale and subscale scores.

##### Compassion for the self

All 371 students completed all SOCS-S items and were included in the CFA. All fit indices showed acceptable fit of five-factor and five-factor hierarchical models, but poor fit of the one-factor model (Table 1). All item loadings in the two five-factor models were significant. All factor intercorrelations in the five-factor model were significant and factor loadings in the five-factor hierarchical model were significant, indicating that the factors are related and elements of an overall self-compassion construct. Based on both the fit indices and significance of factor loadings, the five-factor hierarchical model can be interpreted as providing the best fit. Tables S6 and S4 (see supplementary materials available online) show standardized SOCS-S item and factor loadings, respectively, in the five-factor hierarchical model. Table S7 (see supplementary materials available online) shows correlations between total SOCS-S scale and subscale scores.

#### Internal Consistency

In Table 2, both omega total estimates, calculated using standardized item loadings from five-factor hierarchical models, and Cronbach’s alphas indicate acceptable internal consistency for both the SOCS-O and SOCS-S in this sample.

#### Floor and Ceiling Effects

None of the students received the lowest possible score on the SOCS-O/SOCS-S (20) and 0% and 0.3% received the highest possible score on the SOCS-O and SOCS-S (100), respectively.

#### Interpretability

Table 3 shows the means and standard deviations of total SOCS-O and SOCS-S scores across subgroups of students. As expected, females scored significantly higher on the SOCS-O compared with males, *t*(366) = 2.52, *p* = .012, *d* = 0.39, but there was no significant gender difference in SOCS-S scores, *t*(366) = 0.14, *p* = .891, *d* = 0.02. Length of previous meditation experience had a significant effect on SOCS-S scores only, *F*(3) = 4.35, *p* = .005. Those with 1 to 5 years’ meditation experience scored significantly higher on the SOCS-S compared with those without any meditation experience, *t*(367) = 2.97, *p* = .003, *d* = 0.59. Marital status did not significantly affect scores on the SOCS-O, *F*(1) = 0.35, *p* = .552, or SOCS-S, *F*(1) = 1.88, *p* = .171.

#### Convergent and Discriminant Validity

Table 4 shows correlations between total and subscale scores on the SOCS-O and SOCS-S and other constructs. As predicted, the SOCS-O was significantly correlated with the SCBCS and empathic concern subscale of the IRI, and the SOCS-S was significantly correlated with the SCS-12, at *r* ≥ .50. The SOCS-O was also significantly related to the perspective taking and personal distress subscales of the IRI. Consistent with expectations, the SOCS-S was significantly correlated in expected directions with the FFMQ-15, SWEMWBS, and DASS subscales, with correlations ranging from moderate-large to large in size. We also found significant small-moderate correlations between the SOCS-O and the FFMQ-15 and SWEMWBS, and a small-moderate, significant, negative correlation between the SOCS-O and DASS depression. Altogether, none of the correlations were *r* ≥ .80, at least three quarters of results were consistent with predictions, and at least two correlations were *r* ≥ .50, which supports the convergent and discriminant validity total SOCS-O and SOCS-S scale scores in this student sample.

#### Relationship Between Compassion for the Self and Others

Students scored significantly higher on the SOCS-O compared with the SOCS-S, *t*(370) = 19.23, *p* < .001, *d* = 1.15, 95% confidence interval [1.01, 1.29]. Table S7 (see supplementary materials available online) presents the correlations between total scale and subscale scores on both measures in this sample. Total SOCS-O and SOCS-S scores were found to significantly correlate at *r* = .34 and many SOCS-O and SOCS-S subscales were also significantly correlated. However, the correlation between total scale scores may be artificially inflated given the overlap in wording for universality of suffering items for both scales. We excluded the universality subscale from total SOCS-O and SOCS-S scores and nevertheless found total scores to be significantly correlated at *r* = .20 (*p* < .001).

## Discussion

The aim of this program of research was to develop and evaluate the psychometric properties of two new self-report measures of compassion: the SOCS-O and SOCS-S. Findings from Stages 1 and 2 yielded the 20-item SOCS-O and SOCS-S and findings from Stages 3 and 4 support the factor structures and demonstrate robust psychometric properties of both scales.

For both scales, in both health care staff and student samples, a five-factor hierarchical model, with items loading on respective factors from the five-element compassion definition and factors loading on an overarching compassion factor (Strauss et al., 2016), was found to fit the data well as predicted. Internal consistency of total SOCS-O and SOCS-S scale and subscale items was adequate and the scales showed no indication of floor and ceiling effects. We also facilitated the interpretability of scores on both scales. For example, in both samples, females scored significantly higher on the SOCS-O compared with males, and in health care staff only, SOCS-O scores also significantly differed based on length of previous meditation experience, with those with more meditation experience scoring significantly higher compared with those with little or no meditation experience. In both samples, SOCS-S scores significantly differed based on previous meditation experience, with those with more meditation experience scoring significantly higher compared with those with little or no meditation experience.

The SOCS-O and SOCS-S also showed evidence of convergent and discriminant validity. Consistent with predictions, in both samples, the SOCS-O was significantly correlated with scales measuring compassion for others and empathy and the SOCS-S significantly correlated with an existing Self-Compassion Scale, with correlations large in size, but not so large as to indicate the SOCS-O and SOCS-S are redundant. As hypothesized, the SOCS-S significantly correlated in expected directions with measures of mindfulness, well-being, stress, anxiety, depression, and burnout with correlations ranging from moderate-large to large in size, but not so large as to suggest that they are measuring the same construct. We also found significant small-moderate correlations between the SOCS-O and measures of mindfulness, well-being, and burnout, and significant, small correlations between the SOCS-O and mental health. However, there were key differences between the SOCS-O and SOCS-S in terms of their patterns of association with mental health outcomes. Whereas SOCS-S scale and subscales were in general significantly and negatively correlated with stress, anxiety, and depression, the relationship between the SOCS-O and mental health outcomes was more variable; only the universality and tolerating subscales showed significant, negative relationships which were largely consistent across both samples, and total SOCS-O was significantly correlated with just stress and depression in the health care sample and just depression in the student sample.

Our findings on the relationship between the SOCS-S and related variables support previous research, but current findings on the SOCS-O contrast with previous research which found no relationship between compassion for others and mindfulness, mental health, well-being, and burnout (e.g., Durkin et al., 2016; López et al., 2018; Pommier, 2010). This suggests that the lack of relationship between other-compassion and these constructs may be partly attributable to limitations of existing compassion measures around content validity, item wording, and psychometric properties (Strauss et al., 2016). We also found, for both health care staff and students, a significant and small-moderate to moderate correlation between the SOCS-O and SOCS-S. This is at odds with previous research which at best have found small correlations between self- and other-compassion in nonmeditator and student samples (López et al., 2018; Neff & Pommier, 2013; Pommier, 2010) and at worst found a small-moderate and negative, but nonsignificant, correlation between self- and other-compassion in students (Durkin et al., 2016). Previous findings of little or no empirical overlap between the two may be in part due to issues with the previous measures of compassion used in these studies.

Taken together, current findings support the multidimensional conceptualization of compassion proposed by Strauss et al. (2016) and present the SOCS-O and SOCS-S as new, psychometrically robust self-report measures which overcome limitations of previous compassion scales. Key limitations of previous compassion measures addressed include those relating to content validity (e.g., making sure items are related to suffering and do not tap related constructs), item wording (e.g., making sure items are worded in line with the response scale and frequency terms are omitted), and psychometric properties (e.g., demonstrating adequate internal consistency of scales and subscales and evidence for factor structure).

### Implications

Current findings have theoretical implications for our understanding of compassion constructs, and how they relate to each other and to psychological outcomes, and research and clinical implications. We found that greater compassion for the self and others were related to increased mindfulness and well-being, and decreased burnout, stress, depression, and anxiety. This provides initial support for the cultivation of compassion (e.g., through CBIs) to improve psychological functioning. These findings are particularly valuable given that previous studies have failed to find links between compassion for others and these processes and outcomes (e.g., Durkin et al., 2016; López et al., 2018; Pommier, 2010).

We also found that the same model fit both self-compassion and other-compassion data well, and found small-moderate to moderate, and significant, correlations between the SOCS-O and SOCS-S. This contributes to the discourse on the relationship between self- and other-compassion and has implications for future research examining this association. Our findings are consistent with the notion that compassion refers to a process that can orient both to the self or others and indicate that self- and other-compassion are overlapping constructs. This contrasts to previous research which found little or no empirical overlap between compassion for the self and others (Durkin et al., 2016, López et al., 2018; Neff & Pommier, 2013; Pommier, 2010). Future research should therefore not be deterred by initial findings indicating no relationship between self- and other-compassion and it would be valuable to explore this relationship further.

Moreover, the current program of research addressed an important omission in compassion research and practice by developing valid and reliable measures of compassion. Being able to measure compassion using robust tools is necessary for the growth of this field. We anticipate that these scales will prove valuable in progressing key research areas, including building an evidence base for CBIs by facilitating evaluation of their effectiveness and underlying mechanisms.

### Limitations and Future Research

The SOCS-O and SOCS-S require further testing. Some psychometric properties were not assessed as these were beyond the scope of the current program of research. These include test–retest reliability, sensitivity to change over the course of a CBI or other interventions which would theoretically cultivate compassion, and further tests of convergent and discriminant validity with additional theoretically related and unrelated constructs. Although a total score can be derived from each scale, as these were designed to be multidimensional measures, it would also be important for future studies to examine in greater detail how the five elements of self/other-compassion interact with one another and how they relate independently, and collectively, to outcomes.

Given that compassion is a culturally valued construct, it would be beneficial for future research to examine the extent to which compassion, as measured by the SOCS, overlaps with social desirability, as the basis for taking any social desirability into account when interpreting SOCS scores and findings.

Due to the anonymous nature of the online surveys administered to health care staff in Stages 2 and 3, we were not able to ensure that the two samples were entirely independent. In both Stages, study adverts and survey titles were the same, which would have minimized chances of health care staff completing both sets of surveys. However, future research should try to employ measures to ensure complete independence of validation samples.

Although the current program of research validated the SOCS-O and SOCS-S in health care staff and student samples, research in this field has also recruited from other populations (e.g., clinical populations, meditators, general population) and the scales would benefit from cross-validation in such populations to further support their use and inform understanding of compassion across different groups. Complementing item development through consultation with experts from six continents, and given that the dominant ethnicity of both health care staff and student samples in this study was White and both were U.K. samples, future research should also cross-validate the factor structures of the SOCS-O and SOCS-S in samples from other cultures and countries. As part of this line of research, the compassion scales could be translated into different languages which would enable investigation of research questions such as whether there are cross-cultural differences in the strength of the relationship between self- and other-compassion, and compassion and psychological functioning. Alongside cross-validation of the SOCS-O and SOCS-S in different populations, future studies should also evaluate measurement invariance, and if this holds, latent mean differences across groups. For example, given that the current samples mainly consisted of white females, future studies could assess measurement invariance by gender and culture.

Our findings are consistent with the suggestion that interventions designed to cultivate compassion could improve emotional health outcomes (Kirby et al., 2017). However, we used a cross-sectional design and direction of effects cannot be determined. Future research evaluating the effectiveness of CBIs using the SOCS-O and SOCS-S would provide a more robust test of this possibility. Similarly, the relationship between compassion and burnout is consistent with the observation of diminishing compassion in cases of work-related burnout in the health care sector (Joinson, 1992), although the direction of effect cannot be determined from our findings. Using longitudinal designs would also help address any potential common method variance associated with collecting self-report data from the same respondents at a single point in time (Podsakoff, MacKenzie, Lee, & Podsakoff, 2003).

Self-report methods are not without their limitations and are likely to provide only a partial picture of compassion. It would be beneficial for future research to explore whether the SOCS-O and SOCS-S can be triangulated with nonself-report methods of assessing compassion. For example, baseline SOCS-O and SOCS-S scores and/or change in scores over intervention could be correlated with baseline performance and/or change in performance over intervention on behavioral tasks assessing compassion, such as prosocial games (e.g., the Zurich Prosocial Game; Leiberg, Klimecki, & Singer, 2011). Research which uses both self-report and alternative methods of assessing compassion would also help address any common method variance. However, challenges remain in developing behavioral tasks that can clearly distinguish compassion from distinct but related constructs such as prosocial behavior, empathy, and altruism. With this in mind, the SOCS-O and SOCS-S have the advantage of accessing the private cognitive and emotional motivations that are part of the compassion construct. They may also be helpful in developing and refining behavioral measures which specifically capture compassion.

## Conclusion

Progress in the field of compassion requires robust measures that comprehensively capture compassion for others and compassion for the self. The current program of research developed new theoretically informed and psychometrically robust self-report measures of compassion: the SOCS-O and SOCS-S. Findings support the factor structures of both scales in health care staff and student samples. Both the SOCS-O and SOCS-S consist of the following five subscales which can be seen as elements of an overall self- or other-compassion construct: (a) recognizing suffering, (b) understanding the universality of suffering, (c) feeling for the person suffering and emotionally connecting with their distress, (d) tolerating uncomfortable feelings aroused so that we remain open to and accepting of them in their suffering, and (e) acting or being motivated to act to alleviate suffering. Findings also support the psychometric properties of both scales in terms of their internal consistency, interpretability, floor and ceiling effects, and convergent and discriminant validity. Taken together, the rigorous development process employed in the current research program and emergent psychometric properties of the SOCS-O and SOCS-S support their use in compassion research and practice.

## Supplemental Material

Supplementary_materials_(clean)_r1 – Supplemental material for Development and Psychometric Properties of the Sussex-Oxford Compassion Scales (SOCS) Click here for additional data file. Supplemental material, Supplementary_materials_(clean)_r1 for Development and Psychometric Properties of the Sussex-Oxford Compassion Scales (SOCS) by Jenny Gu, Ruth Baer, Kate Cavanagh, Willem Kuyken and Clara Strauss in Assessment

## Notes

1. The nine measures of compassion reviewed by Strauss et al. (2016) were as follows: Compassionate Love Scale (Sprecher & Fehr, 2005), Santa Clara Brief Compassion Scale (Hwang, Plante, & Lackey, 2008), Martins et al.’s Compassion Scale (Martins, Nicholas, Shaheen, Jones, & Norris, 2013), Self-Compassion Scale (Neff, 2003), Self-Compassion Scale–Short form (Raes et al., 2011), Pommier’s (2010) Compassion Scale, Relational Compassion Scale (Hacker, 2008), Compassionate Care Assessment Tool (Burnell & Agan, 2013), and Schwartz Center Compassionate Care Scale (Lown, Muncer, & Chadwick, 2015).

2. We examined whether the subsample of 115 staff who completed the MBI-HSS differed in terms of demographic variables (age, gender, ethnicity, marital status, level of education, and job role) from the rest of the Stage 3 sample. The only significant difference found was for level of education, χ^2^(4) = 16.62, *p* = .002; in the subsample, 1 (0.9% [1.1%]) had no formal qualifications, 14 (12.6% [12.7%]) had some GCSEs (U.K. school qualifications received at age 16) or equivalent qualifications, 22 (19.8% [17.5%]) had some A Levels (U.K. school qualifications received at age 18) or equivalent qualifications, 32 (28.8% [46.0%]) had a bachelor’s degree or equivalent, and 42 (37.8% [22.6%]) had a higher degree, such as a master’s or doctoral degree. Percentages given in square brackets are for those in the rest of the sample.

## References

[bibr1-1073191119860911] Akaike H. (1974). A new look at the statistical model identification. IEEE Transactions on Automatic Control, 19, 716-723.

[bibr2-1073191119860911] American Medical Association. (2001). AMA principles of medical ethics. Retrieved from www.ama-assn.org/sites/default/files/media-browser/principles-of-medical-ethics.pdf

[bibr3-1073191119860911] Baer R. A. Carmody J. Hunsinger M. (2012). Weekly change in mindfulness and perceived stress in a mindfulness-based stress reduction program. Journal of Clinical Psychology, 68, 755-765. 22623334 10.1002/jclp.21865

[bibr4-1073191119860911] Baer R. A. Smith G. T. Hopkins J. Krietemeyer J. Toney L. (2006). Using self-report assessment methods to explore facets of mindfulness. Assessment, 13, 27-45. 16443717 10.1177/1073191105283504

[bibr5-1073191119860911] Barker C. Pistrang N. Elliott R. (2002). Research methods in clinical psychology. London, England: Wiley.

[bibr6-1073191119860911] Bentler P. M. (1990). Comparative fit indexes in structural models. Psychological Bulletin, 107, 238-246. 2320703 10.1037/0033-2909.107.2.238

[bibr7-1073191119860911] Bentler P. M. Bonett D. G. (1980). Significance tests and goodness of fit in the analysis of covariance structures. Psychological Bulletin, 88, 588-606.

[bibr8-1073191119860911] Burnell L. Agan D. L. (2013). Compassionate care: Can it be defined and measured? The development of the Compassionate Care Assessment Tool. International Journal of Caring Sciences, 6, 180-187.

[bibr9-1073191119860911] Byrne B. M. (2005). Factor analytic models: Viewing the structure of an assessment instrument from three perspectives. Journal of Personality Assessment, 85, 17-32. 16083381 10.1207/s15327752jpa8501_02

[bibr10-1073191119860911] Chen F. Curran P. J. Bollen K. A. Kirby J. Paxton P. (2008). An empirical evaluation of the use of fixed cutoff points in RMSEA test statistic in structural equation models. Sociological Methods & Research, 36, 462-494. 19756246 10.1177/0049124108314720 PMC2743032

[bibr11-1073191119860911] Compassion in Education Foundation. (2016). Bringing compassion into education and learning: The Third Strategic Plan and Progress Report. Retrieved from www.coedfoundation.org.uk/pdfs/CoEd_Foundation_strategic_plan.pdf

[bibr12-1073191119860911] Costello A. B. Osborne J. W. (2005). Best practices in exploratory factor analysis: Four recommendations for getting the most from your analysis. Practical Assessment, Research & Evaluation, 10(7), 1-9.

[bibr13-1073191119860911] Dahlsgaard K. Peterson C. Seligman M. E. (2005). Shared virtue: The convergence of valued human strengths across culture and history. Review of General Psychology, 9, 203-213.

[bibr14-1073191119860911] Dalai Lama. (2002). How to practice: The way to a meaningful life. New York, NY: Pocket Books.

[bibr15-1073191119860911] Darwin C. (1871). The descent of man, and selection in relation to sex. London, England: John Murray.

[bibr16-1073191119860911] Davidson R. J. Schuyler B. S. (2015). Neuroscience of happiness. In Helliwell J. F. Layard R. Sachs J. (Eds.), World happiness report (pp. 88-105). New York, NY: Sustainable Development Solutions Network Retrieved from http://aeidl.eu/images/stories/pdf/worldhappinessreport2015.pdf

[bibr17-1073191119860911] Davis M. H. (1980). A multidimensional approach to individual differences in empathy. Retrieved from https://www.uv.es/~friasnav/Davis_1980.pdf

[bibr18-1073191119860911] Department of Health. (2013). The handbook to the NHS constitution. Retrieved from www.gov.uk/government/publications/the-nhs-constitution-for-england

[bibr19-1073191119860911] de Waal F. (2009). The age of empathy. New York, NY: Harmony.

[bibr20-1073191119860911] DeVellis R. F. (2016). Scale development: Theory and applications (Vol. 26). Thousand Oaks, CA: Sage.

[bibr21-1073191119860911] Durkin M. Beaumont E. Martin C. J. H. Carson J. (2016). A pilot study exploring the relationship between self-compassion, self-judgement, self-kindness, compassion, professional quality of life and wellbeing among UK community nurses. Nurse Education Today, 46, 109-114. doi:10.1016/j.nedt.2016.08.030 27621200

[bibr22-1073191119860911] Epstein R. M. Franks P. Shields C. G. Meldrum S. C. Miller K. N. Campbell T. L. Fiscella K. (2005). Patient-centered communication and diagnostic testing. Annals of Family Medicine, 3, 415-421. 16189057 10.1370/afm.348 PMC1466928

[bibr23-1073191119860911] Feldman C. Kuyken W. (2011). Compassion in the landscape of suffering. Contemporary Buddhism, 12, 143-155.

[bibr24-1073191119860911] Field A. (2013). Discovering statistics using IBM SPSS statistics (4th ed.). Thousand Oaks, CA: Sage.

[bibr25-1073191119860911] Furr M. (2011). Scale construction and psychometrics for social and personality psychology. Thousand Oaks, CA: Sage.

[bibr26-1073191119860911] Gilbert P. (2005). Compassion and cruelty: A biopsychosocial approach. In: Gilbert P. (Ed.), Compassion: Conceptualisations, research and use in psychotherapy (pp. 9-74). London, England: Routledge.

[bibr27-1073191119860911] Gilbert P. (2009). Introducing compassion-focused therapy. Advances in Psychiatric Treatment, 15, 199-208.

[bibr28-1073191119860911] Gilbert P. (2010). The compassionate mind. London, England: Constable & Robinson.

[bibr29-1073191119860911] Gilbert P. (2014). The origins and nature of compassion focused therapy. British Journal of Clinical Psychology, 53, 6-41. 24588760 10.1111/bjc.12043

[bibr30-1073191119860911] Gu J. Cavanagh K. Baer R. Strauss C. (2017). An empirical examination of the factor structure of compassion. PLoS ONE, 12, e0172471. 28212391 10.1371/journal.pone.0172471 PMC5315311

[bibr31-1073191119860911] Gu J. Strauss C. Crane C. Barnhofer T. Karl A. Cavanagh K. Kuyken W. (2016). Examining the factor structure of the 39-item and 15-item versions of the Five Facet Mindfulness Questionnaire before and after mindfulness-based cognitive therapy for people with recurrent depression. Psychological Assessment, 28, 791-802. 27078186 10.1037/pas0000263 PMC4928699

[bibr32-1073191119860911] Hacker T. (2008). The relational compassion scale: Development and validation of a new self-rated scale for the assessment of self-other compassion (Doctoral dissertation). Retrieved from http://theses.gla.ac.uk/462/1/2008hackerDclinpsy.pdf

[bibr33-1073191119860911] Henry J. D. Crawford J. R. (2005). The short-form version of the Depression Anxiety Stress Scales (DASS-21): Construct validity and normative data in a large non-clinical sample. British Journal of Clinical Psychology, 44, 227-239. 16004657 10.1348/014466505X29657

[bibr34-1073191119860911] Hinkin T. R. (1998). A brief tutorial on the development of measures for use in survey questionnaires. Organizational Research Methods, 1(1), 104-121.

[bibr35-1073191119860911] Hu L. T. Bentler P. M. (1999). Cutoff criteria for fit indexes in covariance structure analysis: Conventional criteria versus new alternatives. Structural Equation Modeling: A Multidisciplinary Journal, 6, 1-55.

[bibr36-1073191119860911] Hwang J. Y. Plante T. Lackey K. (2008). The development of the Santa Clara brief compassion scale: An abbreviation of Sprecher and Fehr’s compassionate love scale. Pastoral Psychology, 56, 421-428.

[bibr37-1073191119860911] IBM. (2016). IBM SPSS Statistics for Windows (Version 24.0) [Computer software]. Armonk, NY: Author.

[bibr38-1073191119860911] Jazaieri H. Jinpa G. T. McGonigal K. Rosenberg E. L. Finkelstein J. Simon-Thomas E.. . . Goldin P. R. (2013). Enhancing compassion: A randomized controlled trial of a compassion cultivation training program. Journal of Happiness Studies, 14, 1113-1126.

[bibr39-1073191119860911] Joinson C. (1992). Coping with compassion fatigue. Nursing, 22, 116-118. 1570090

[bibr40-1073191119860911] Kabat-Zinn J. (1982). An outpatient program in behavioral medicine for chronic pain patients based on the practice of mindfulness meditation: Theoretical considerations and preliminary results. General Hospital Psychiatry, 4, 33-47. 7042457 10.1016/0163-8343(82)90026-3

[bibr41-1073191119860911] Kanov J. M. Maitlis S. Worline M. C. Dutton J. E. Frost P. J. Lilius J. M. (2004). Compassion in organizational life. American Behavioral Scientist, 47, 808-827.

[bibr42-1073191119860911] Kirby J. N. Tellegen C. L. Steindl S. R. (2017). A meta-analysis of compassion-based interventions: Current state of knowledge and future directions. Behavior Therapy, 48, 778-792. 29029675 10.1016/j.beth.2017.06.003

[bibr43-1073191119860911] Kline P. (1999). The handbook of psychological testing (2nd ed.). London, England: Routledge.

[bibr44-1073191119860911] Kline R. B. (2015). Principles and practice of structural equation modelling (4th ed.). New York, NY: Guilford Press.

[bibr45-1073191119860911] Leiberg S. Klimecki O. Singer T. (2011). Short-term compassion training increases prosocial behavior in a newly developed prosocial game. PLoS ONE, 6, e17798. 21408020 10.1371/journal.pone.0017798 PMC3052380

[bibr46-1073191119860911] Levine T. R. (2005). Confirmatory factor analysis and scale validation in communication research. Communication Research Reports, 22, 335-338.

[bibr47-1073191119860911] López A. Sanderman R. Ranchor A. V. Schroevers M. J. (2018). Compassion for others and self-compassion: Levels, correlates, and relationship with psychological well-being. Mindfulness, 9, 325-331. 29387268 10.1007/s12671-017-0777-z PMC5770484

[bibr48-1073191119860911] Lown B. A. Muncer S. J. Chadwick R. (2015). Can compassionate healthcare be measured? The Schwartz Center Compassionate Care Scale™. Patient Education and Counseling, 98, 1005-1010. 25921380 10.1016/j.pec.2015.03.019

[bibr49-1073191119860911] Marsh H. W. Hau K. T. Wen Z. (2004). In search of golden rules: Comment on hypothesis-testing approaches to setting cutoff values for fit indexes and dangers in overgeneralizing Hu and Bentler’s (1999) findings. Structural Equation Modeling, 11, 320-341.

[bibr50-1073191119860911] Martins D. Nicholas N. A. Shaheen M. Jones L. Norris K. (2013). The development and evaluation of a compassion scale. Journal of Health Care for the Poor and Underserved, 24, 1235-1246. 23974394 10.1353/hpu.2013.0148 PMC3915801

[bibr51-1073191119860911] Maslach C. Jackson S. E. Leiter M. P. (1981). Maslach Burnout Inventory: MBI. Palo Alto, CA: Consulting Psychologists press.

[bibr52-1073191119860911] Matsunaga M. (2010). How to factor-analyze your data right: Do’s, don’ts, and how-to’s. International Journal of Psychological Research, 3, 97-110.

[bibr53-1073191119860911] McNeish D. (2017). Thanks coefficient alpha, we’ll take it from here. Psychological Methods, 23, 412-433. doi:10.1037/met0000144 28557467

[bibr54-1073191119860911] Mongrain M. Chin J. M. Shapira L. B. (2011). Practicing compassion increases happiness and self-esteem. Journal of Happiness Studies, 12, 963-981.

[bibr55-1073191119860911] Muthén L. K. Muthén B. O. (1998-2015). Mplus user’s guide (7th ed.). Los Angeles, CA: Muthén & Muthén.

[bibr56-1073191119860911] Neff K. D. (2003). The development and validation of a scale to measure self-compassion. Self and Identity, 2, 223-250.

[bibr57-1073191119860911] Neff K. D. Germer C. K. (2013). A pilot study and randomized controlled trial of the mindful self-compassion program. Journal of Clinical Psychology, 69, 28-44. 23070875 10.1002/jclp.21923

[bibr58-1073191119860911] Neff K. D. Pommier E. (2013). The relationship between self-compassion and other-focused concern among college undergraduates, community adults, and practicing meditators. Self and Identity, 12, 160-176.

[bibr59-1073191119860911] NHS England. (2017). NHS staff health and wellbeing. Retrieved from https://www.england.nhs.uk/wp-content/uploads/2018/05/staff-health-wellbeing-cquin-2017-19-implementation-support.pdf

[bibr60-1073191119860911] Norko M. A. (2005). Commentary: Compassion at the core of forensic ethics. Journal of the American Academy of Psychiatry and the Law, 33, 386-389. 16186205

[bibr61-1073191119860911] Podsakoff P. M. MacKenzie S. B. Lee J. Y. Podsakoff N. P. (2003). Common method biases in behavioral research: A critical review of the literature and recommended remedies. Journal of Applied Psychology, 88, 879-903. 14516251 10.1037/0021-9010.88.5.879

[bibr62-1073191119860911] Pommier E. A. (2010). The compassion scale (Doctoral dissertation). Retrieved from https://self-compassion.org/wp-content/uploads/2015/02/POMMIER-DISSERTATION-compassion.pdf

[bibr63-1073191119860911] Raes F. Pommier E. Neff K. D. Van Gucht D. (2011). Construction and factorial validation of a short form of the self-compassion scale. Clinical Psychology & Psychotherapy, 18, 250-255. 21584907 10.1002/cpp.702

[bibr64-1073191119860911] Sanghavi D. M. (2006). What makes for a compassionate patient-caregiver relationship? Joint Commission Journal on Quality and Patient Safety, 32, 283-292. 16761793 10.1016/s1553-7250(06)32037-5

[bibr65-1073191119860911] Schermelleh-Engel K. Moosbrugger H. Müller H. (2003). Evaluating the fit of structural equation models: Tests of significance and descriptive goodness-of-fit measures. Methods of Psychological Research Online, 8(2), 23-74.

[bibr66-1073191119860911] Segal Z. V. Williams J. M. Teasdale J. (2002). Mindfulness-based cognitive therapy for depression: A new approach to preventing relapse. New York, NY: Guilford Press.

[bibr67-1073191119860911] Segal Z. V. Williams J. M. Teasdale J. (2013). Mindfulness-based cognitive therapy for depression (2nd ed.). New York, NY: Guilford Press.

[bibr68-1073191119860911] Sprecher S. Fehr B. (2005). Compassionate love for close others and humanity. Journal of Social and Personal Relationships, 22, 629-651.

[bibr69-1073191119860911] Steiger J. H. (1990). Structural model evaluation and modification: An interval estimation approach. Multivariate Behavioral Research, 25, 173-180. 26794479 10.1207/s15327906mbr2502_4

[bibr70-1073191119860911] Stewart-Brown S. Tennant A. Tennant R. Platt S. Parkinson J. Weich S. (2009). Internal construct validity of the Warwick-Edinburgh Mental Well-Being Scale (WEMWBS): A Rasch analysis using data from the Scottish Health Education Population Survey. Health and quality of life outcomes, 7, 15. doi:10.1186/1477-7525-7-15 19228398 PMC2669062

[bibr71-1073191119860911] Strauss C. Taylor B. L. Gu J. Kuyken W. Baer R. Jones F. Cavanagh K. (2016). What is compassion and how can we measure it? A review of definitions and measures. Clinical Psychology Review, 47, 15-27. 27267346 10.1016/j.cpr.2016.05.004

[bibr72-1073191119860911] Terwee C. B. Bot S. D. M. de Boer M. R. van der Windt D. A. W. M. Knol D. L. Dekker J.. . . de Vet H. C. W. (2007). Quality criteria were proposed for measurement properties of health status questionnaires. Journal of Clinical Epidemiology, 60, 34-42. 17161752 10.1016/j.jclinepi.2006.03.012

[bibr73-1073191119860911] Williams M. J. Dalgleish T. Karl A. Kuyken W. (2014). Examining the factor structures of the Five-Facet Mindfulness Questionnaire and the Self-Compassion Scale. Psychological Assessment, 26, 407-418. 24490681 10.1037/a0035566

[bibr74-1073191119860911] Wispe L. (1991). The psychology of sympathy. New York, NY: Plenum Press.

