# Supplementary material for: Development and Psychometric Properties of the Sussex-Oxford Compassion Scales (SOCS)
Source: Assessment. 2019 Jul 29;27(1):3–20. doi: 10.1177/1073191119860911 (PMC6906538; doi:10.1177/1073191119860911)
Supplement: Supplementary_materials_(clean)_r1 – Supplemental material for Development and Psychometric Properties of the Sussex-Oxford Compassion Scales (SOCS) [file Supplementary_materials_(clean)_r1.pdf]

## **Supplementary Materials** for:

## Development and Psychometric Properties of the Sussex-Oxford Compassion Scales (SOCS)

Table S1

*Standardised loadings of compassion for others items on each factor in a sample of 932 healthcare staff (Stage 2)*

| | Standardised loading | *SE* |
|---|---|---|
| **Recognising suffering** | | |
| *I notice when others are feeling distressed. | 0.826 | 0.02 |
| *I recognise when other people are feeling distressed without them having to tell me. | 0.799 | 0.02 |
| *I recognise signs of suffering in others. | 0.797 | 0.02 |
| *I'm quick to notice early signs of distress in others. | 0.780 | 0.02 |
| Even if I'm busy, I can still tell when other people are upset. | 0.770 | 0.02 |
| I can tell when someone else is holding back tears. | 0.696 | 0.02 |
| I recognise when other people first start feeling upset. | 0.689 | 0.03 |
| I can tell when someone else is pretending to be fine when they're not. | 0.672 | 0.02 |
| I'm better than most people at recognising when others are struggling emotionally. | 0.647 | 0.03 |
| It takes me a while to notice if someone is upset about something. (negatively phrased) | 0.498 | 0.04 |
| I misjudge how others are feeling. (negatively phrased) | 0.489 | 0.03 |
| I'm unaware of other people's distress. (negatively phrased) | 0.173 | 0.04 |
| **Understanding the universality of suffering** | | |
| *I understand that everyone experiences suffering at some point in their lives. | 0.872 | 0.01 |
| *I understand that feeling upset at times is part of human nature. | 0.851 | 0.02 |
| *Like me, I know that other people also experience struggles in life. | 0.811 | 0.02 |
| *I know that we can all feel upset at times when we are wronged. | 0.794 | 0.02 |
| I believe that feeling low at times is part of being human. | 0.772 | 0.02 |
| I know that we can all feel distressed when things don't go well in our lives. | 0.768 | 0.02 |
| I understand that experiences of suffering are shared by everybody. | 0.754 | 0.02 |
| I know that everyone has ups and downs in their lives. | 0.673 | 0.03 |
| I believe that no matter how different people are, we all experience difficulties in life. | 0.672 | 0.03 |
| I know that I'm not alone in experiencing suffering. | 0.641 | 0.03 |
| I believe that suffering is a shared human experience. | 0.608 | 0.02 |
| When someone else is going through a hard time, I can understand and relate to their experience. | 0.355 | 0.03 |
| **Feeling for the person suffering** | | |
| *When someone is going through a difficult time, I feel kindly towards them. | 0.769 | 0.02 |

| | | |
|---|---|---|
| *When I hear about bad things happening to other people, I feel concern for their wellbeing. | 0.707 | 0.03 |
| *When someone is upset, I try to tune in to how they're feeling. | 0.688 | 0.02 |
| *I'm sensitive to other people's distress. | 0.682 | 0.03 |
| Even if I don't like someone, I can feel warmly towards them when they're in distress. | 0.638 | 0.03 |
| I'm emotionally distant from others when they're upset. (negatively phrased) | 0.557 | 0.03 |
| I feel indifferent towards other people's struggles. (negatively phrased) | 0.512 | 0.04 |
| I feel emotionally detached from other people's problems. (negatively phrased) | 0.472 | 0.04 |
| **Tolerating uncomfortable feelings** | | |
| *When someone else is upset, I try to stay open to their feelings rather than avoid them. | 0.750 | 0.03 |
| *I stay with and listen to other people when they're upset even if it's hard to bear. | 0.707 | 0.03 |
| *I connect with the suffering of others without judging them. | 0.621 | 0.04 |
| *When someone else is upset, I can be there for them without feeling overwhelmed by their distress. | 0.437 | 0.06 |
| I can stay with uncomfortable feelings in response to other people's distress. | 0.383 | 0.04 |
| I connect with other people's distress without letting it overwhelm me. | 0.378 | 0.06 |
| I can be around someone who is upset without letting their distress take over me. | 0.367 | 0.06 |
| When others are upset, I feel so uncomfortable that I'm unable to be around them. (negatively phrased) | 0.343 | 0.04 |
| I feel overwhelmed by other people's problems. (negatively phrased) | 0.204 | 0.05 |
| If I feel uncomfortable in response to someone else's distress, I don't let myself get carried away by my feelings. | 0.204 | 0.05 |
| **Acting or being motivated to act to alleviate suffering** | | |
| *When others are struggling, I try to do things that would be helpful. | 0.823 | 0.02 |
| *When someone is going through a difficult time, I try to look after them. | 0.815 | 0.02 |
| *When I see someone in need, I try to do what's best for them. | 0.806 | 0.02 |
| *When I see that someone is upset, I do my best to take care of them. | 0.805 | 0.02 |
| If someone is suffering, I try to do something helpful to reduce their distress. | 0.800 | 0.02 |
| I try to help people feel better when they are distressed, even if I can't do anything about the problem. | 0.790 | 0.02 |
| When someone is going through a hard time, I try to do what's best for them. | 0.726 | 0.02 |
| I try to prioritise the needs of others when they're facing difficulties. | 0.690 | 0.02 |
| When others are feeling down, I think of ways I can make things better for them. | 0.685 | 0.02 |
| I think of useful ways I can help people when they're struggling. | 0.664 | 0.03 |
| If someone looks troubled, I stop and ask if there's anything I can do. | 0.622 | 0.02 |
| I'm drawn to helping those in need. | 0.597 | 0.03 |
| I'm dismissive of other people's difficulties and problems. (negatively phrased) | 0.487 | 0.04 |

| | | |
|---|---|---|
| I go easy on other people when they're feeling upset. | 0.466 | 0.04 |
| When others are going through a difficult time, I leave them to deal with their own problems. (negatively phrased) | 0.431 | 0.04 |

*Note*. Items preceded by an asterisk indicate ones which were selected for inclusion in the SOCS-O. All standardised loadings were significant ($p < .001$). Standardised loadings are reported to three decimal places to facilitate selection of the highest loading items.

Table S2

*Standardised loadings of compassion for the self items on each factor in a sample of 947 healthcare staff (Stage 2)*

| | Standardised loading | *SE* |
|---|---|---|
| **Recognising suffering** | | |
| *I'm quick to notice early signs of distress in myself. | 0.833 | 0.01 |
| *I notice when I'm feeling distressed. | 0.833 | 0.01 |
| *I'm good at recognising when I'm feeling distressed. | 0.778 | 0.02 |
| *I recognise signs of suffering in myself. | 0.773 | 0.02 |
| Even if I'm busy, I can still tell when I'm upset. | 0.713 | 0.02 |
| Compared to most people, I'm better at recognising when I'm struggling emotionally. | 0.657 | 0.02 |
| I recognise when I first start feeling upset. | 0.656 | 0.02 |
| It takes me a while to notice if I'm upset about something. (negatively phrased) | 0.510 | 0.03 |
| I can tell when I'm holding back tears. | 0.504 | 0.03 |
| I can tell when I'm pretending to be fine when I'm not. | 0.488 | 0.03 |
| I'm unaware of my own distress. (negatively phrased) | 0.377 | 0.04 |
| **Understanding the universality of suffering** | | |
| *I understand that everyone experiences suffering at some point in their lives. | 0.873 | 0.02 |
| *Like me, I know that other people also experience struggles in life. | 0.858 | 0.01 |
| *I understand that feeling upset at times is part of human nature. | 0.842 | 0.02 |
| *I know that we can all feel distressed when things don't go well in our lives. | 0.802 | 0.02 |
| I understand that experiences of suffering are shared by everybody. | 0.802 | 0.03 |
| I know that we can all feel upset at times when we are wronged. | 0.790 | 0.02 |
| I know that everyone has ups and downs in their lives. | 0.735 | 0.02 |
| I believe that feeling low at times is part of being human. | 0.734 | 0.02 |
| I believe that no matter how different people are, we all experience difficulties in life. | 0.678 | 0.03 |
| I believe that suffering is a shared human experience. | 0.641 | 0.02 |
| I know that I'm not alone in experiencing suffering. | 0.615 | 0.03 |
| When I'm going through a hard time, I take comfort in knowing that we all go through similar experiences. | 0.388 | 0.03 |
| **Feeling for the person suffering** | | |
| *When I'm going through a difficult time, I feel kindly towards myself. | 0.859 | 0.01 |
| *When bad things happen to me, I feel caring towards myself. | 0.818 | 0.02 |
| *Even when I'm disappointed with myself, I can feel warmly towards myself when I'm in distress. | 0.783 | 0.02 |
| *When I'm upset, I try to tune in to how I'm feeling. | 0.652 | 0.03 |

| | | |
|---|---|---|
| I'm sensitive to my own distress. | 0.510 | 0.04 |
| I feel indifferent towards my own struggles. (negatively phrased) | 0.402 | 0.04 |
| I block out my feelings when I'm upset. (negatively phrased) | 0.375 | 0.04 |
| I feel emotionally detached from my own problems. (negatively phrased) | 0.204 | 0.04 |
| **Tolerating uncomfortable feelings** | | |
| *When I'm upset, I try to stay open to my feelings rather than avoid them. | 0.718 | 0.03 |
| *I connect with my own suffering without judging myself. | 0.716 | 0.02 |
| *When I'm upset, I can let the emotions be there without feeling overwhelmed. | 0.687 | 0.03 |
| *I connect with my own distress without letting it overwhelm me. | 0.684 | 0.03 |
| I face my own feelings when I'm upset even if it's hard to bear. | 0.668 | 0.03 |
| I can be upset without letting my feelings take over me. | 0.651 | 0.03 |
| I'm able to tolerate uncomfortable feelings when I'm distressed. | 0.608 | 0.03 |
| I feel overwhelmed by my own problems. (negatively phrased) | 0.601 | 0.03 |
| When I'm in distress, I allow myself to experience the emotions as they are, even if they're uncomfortable. | 0.589 | 0.03 |
| When I'm upset, I feel so uncomfortable that I push the feelings away. (negatively phrased) | 0.539 | 0.03 |
| I'm judgemental of myself when I'm going through a hard time. (negatively phrased) | 0.487 | 0.04 |
| I overreact to my own problems. (negatively phrased) | 0.486 | 0.03 |
| **Acting or being motivated to act to alleviate suffering** | | |
| *When I'm upset, I try to do what's best for myself. | 0.867 | 0.01 |
| *When I'm going through a difficult time, I try to look after myself. | 0.866 | 0.01 |
| *I try to make myself feel better when I'm distressed, even if I can't do anything about the cause. | 0.848 | 0.01 |
| *When I'm upset, I do my best to take care of myself. | 0.846 | 0.01 |
| When I'm struggling, I try to do things that would be helpful. | 0.837 | 0.01 |
| When I'm going through a hard time, I try to do what's best for myself. | 0.799 | 0.02 |
| If I'm suffering, I try to do something helpful to reduce my distress. | 0.787 | 0.02 |
| I feel a desire to help myself when I'm in need. | 0.783 | 0.02 |
| When I feel troubled, I stop to ask myself what I could do that might help. | 0.732 | 0.02 |
| I go easy on myself when I'm feeling upset. | 0.712 | 0.02 |
| I think of useful ways I can help myself when I'm struggling. | 0.707 | 0.02 |
| I try to prioritise my own needs when I'm facing difficulties. | 0.692 | 0.02 |
| When I'm feeling down, I think of ways I can make things better for myself. | 0.634 | 0.03 |
| When I'm going through a difficult time, I can be hard on myself. (negatively phrased) | 0.516 | 0.03 |
| I'm dismissive of my own difficulties and problems. (negatively phrased) | 0.490 | 0.03 |

*Note.* Items preceded by an asterisk indicate ones which were selected for inclusion in the SOCS-S. All standardised loadings were significant ($p < .001$). Standardised loadings are reported to three decimal places to facilitate selection of the highest loading items.

Table S3

*Standardised item loadings for the compassion for others five-factor hierarchical model in both validation samples (Stages 3 and 4)*

| | 1,242 healthcare staff (Stage 3) | 371 students (Stage 4) |
|---|---|---|
| **Recognising suffering** | | |
| I notice when others are feeling distressed. | 0.88 (0.01)** | 0.83 (0.02)** |
| I recognise when other people are feeling distressed without them having to tell me. | 0.78 (0.02)** | 0.76 (0.04)** |
| I recognise signs of suffering in others. | 0.82 (0.01)** | 0.70 (0.04)** |
| I'm quick to notice early signs of distress in others. | 0.83 (0.01)** | 0.82 (0.02)** |
| **Understanding the universality of suffering** | | |
| I understand that everyone experiences suffering at some point in their lives. | 0.90 (0.01)** | 0.84 (0.03)** |
| I understand that feeling upset at times is part of human nature. | 0.89 (0.01)** | 0.86 (0.03)** |
| Like me, I know that other people also experience struggles in life. | 0.85 (0.02)** | 0.81 (0.04)** |
| I know that we can all feel upset at times when we are wronged. | 0.81 (0.02)** | 0.77 (0.04)** |
| **Feeling for the person suffering** | | |
| When someone is going through a difficult time, I feel kindly towards them. | 0.72 (0.14)** | 0.66 (0.06)** |
| When I hear about bad things happening to other people, I feel concern for their wellbeing. | 0.70 (0.02)** | 0.58 (0.05)** |
| When someone is upset, I try to tune in to how they're feeling. | 0.69 (0.02)** | 0.69 (0.03)** |
| I'm sensitive to other people's distress. | 0.73 (0.02)** | 0.60 (0.05)** |
| **Tolerating uncomfortable feelings** | | |
| When someone else is upset, I try to stay open to their feelings rather than avoid them. | 0.77 (0.02)** | 0.75 (0.04)** |
| I stay with and listen to other people when they're upset even if it's hard to bear. | 0.72 (0.02)** | 0.70 (0.04)** |
| I connect with the suffering of others without judging them. | 0.71 (0.02)** | 0.56 (0.06)** |
| When someone else is upset, I can be there for them without feeling overwhelmed by their distress. | 0.42 (0.03)** | 0.17 (0.06)* |
| **Acting or being motivated to act to alleviate suffering** | | |
| When others are struggling, I try to do things that would be helpful. | 0.84 (0.02)** | 0.76 (0.03)** |
| When someone is going through a difficult time, I try to look after them. | 0.87 (0.01)** | 0.82 (0.03)** |
| When I see someone in need, I try to do what's best for them. | 0.83 (0.01)** | 0.76 (0.03)** |
| When I see that someone is upset, I do my best to take care of them. | 0.82 (0.01)** | 0.75 (0.03)** |

Standard errors are given in parentheses. ** $p < .001$, * $p < .01$.

Table S4

*Standardised loadings of factors to an overall compassion factor in the five-factor hierarchical model in both validation samples (Stages 3 and 4)*

| | Compassion for others | | Compassion for the self | |
|---|---|---|---|---|
| | 1,242 healthcare staff (Stage 3) | 371 students (Stage 4) | 1,216 healthcare staff (Stage 3) | 371 students (Stage 4) |
| Recognising suffering | 0.78 (0.02)* | 0.62 (0.05)* | 0.59 (0.03)* | 0.33 (0.06)* |
| Understanding the universality of suffering | 0.58 (0.03)* | 0.42 (0.06)* | 0.34 (0.03)* | 0.26 (0.05)* |
| Feeling for the person suffering | 0.98 (0.01)* | 0.92 (0.04)* | 0.99 (0.01)* | 0.98 (0.02)* |
| Tolerating uncomfortable feelings | 0.93 (0.02)* | 0.91 (0.03)* | 0.94 (0.01)* | 0.87 (0.03)* |
| Acting or being motivated to act to alleviate suffering | 0.92 (0.01)* | 0.95 (0.03)* | 0.94 (0.01)* | 0.94 (0.02)* |

Standard errors are given in parentheses. * $p < .001$.

Table S5

*Correlation coefficients between total scale and subscale scores on the SOCS-O and SOCS-S using available data from 1,319 healthcare staff (Stage 3)*

| | SOCS-O | 1 | 2 | 3 | 4 | 5 | SOCS-S | 6 | 7 | 8 | 9 | 10 |
|---|---|---|---|---|---|---|---|---|---|---|---|---|
| **Sussex-Oxford Compassion for Others Scale** (SOCS-O) | - | | | | | | | | | | | |
| 1. Recognising suffering | .80* | - | | | | | | | | | | |
| 2. Understanding the universality of suffering | .70* | .41* | - | | | | | | | | | |
| 3. Feeling for the person suffering | .88* | .66* | .49* | - | | | | | | | | |
| 4. Tolerating uncomfortable feelings | .84* | .61* | .48* | .68* | - | | | | | | | |
| 5. Acting or being motivated to act to alleviate suffering | .87* | .64* | .47* | .77* | .71* | - | | | | | | |
| **Sussex-Oxford Compassion for the Self Scale** (SOCS-S) | .40* | .33* | .39* | .32* | .37* | .25* | - | | | | | |
| 6. Recognising suffering | .37* | .36* | .33* | .30* | .31* | .25* | .74* | - | | | | |
| 7. Understanding the universality of suffering | .51* | .27* | .78* | .36* | .36* | .32* | .55* | .41* | - | | | |
| 8. Feeling for the person suffering | .25* | .23* | .20* | .23* | .25* | .15* | .89* | .52* | .30* | - | | |
| 9. Tolerating uncomfortable feelings | .25* | .22* | .17* | .19* | .32* | .15* | .83* | .46* | .29* | .75* | - | |
| 10. Acting or being motivated to act to alleviate suffering | .23* | .22* | .16* | .19* | .24* | .15* | .88* | .52* | .28* | .84* | .72* | - |

*$p < .001$.

Table S6

*Standardised item loadings for the compassion for the self five-factor hierarchical model in both validation samples (Stages 3 and 4)*

| | 1,216 healthcare staff (Stage 3) | 371 students (Stage 4) |
|---|---|---|
| **Recognising suffering** | | |
| I'm quick to notice early signs of distress in myself. | 0.85 (0.01)* | 0.80 (0.03)* |
| I notice when I'm feeling distressed. | 0.81 (0.02)* | 0.81 (0.03)* |
| I'm good at recognising when I'm feeling distressed. | 0.80 (0.02)* | 0.77 (0.03)* |
| I recognise signs of suffering in myself. | 0.76 (0.02)* | 0.69 (0.04)* |
| **Understanding the universality of suffering** | | |
| I understand that everyone experiences suffering at some point in their lives. | 0.88 (0.02)* | 0.91 (0.02)* |
| Like me, I know that other people also experience struggles in life. | 0.86 (0.02)* | 0.87 (0.03)* |
| I understand that feeling upset at times is part of human nature. | 0.87 (0.02)* | 0.85 (0.03)* |
| I know that we can all feel distressed when things don't go well in our lives. | 0.84 (0.02)* | 0.77 (0.04)* |
| **Feeling for the person suffering** | | |
| When I'm going through a difficult time, I feel kindly towards myself. | 0.85 (0.01)* | 0.87 (0.02)* |
| When bad things happen to me, I feel caring towards myself. | 0.79 (0.02)* | 0.87 (0.02)* |
| Even when I'm disappointed with myself, I can feel warmly towards myself when I'm in distress. | 0.77 (0.02)* | 0.76 (0.03)* |
| When I'm upset, I try to tune in to how I'm feeling. | 0.65 (0.02)* | 0.54 (0.05)* |
| **Tolerating uncomfortable feelings** | | |
| When I'm upset, I try to stay open to my feelings rather than avoid them. | 0.69 (0.02)* | 0.47 (0.06)* |
| I connect with my own suffering without judging myself. | 0.72 (0.02)* | 0.79 (0.03)* |
| When I'm upset, I can let the emotions be there without feeling overwhelmed. | 0.57 (0.03)* | 0.63 (0.05)* |
| I connect with my own distress without letting it overwhelm me. | 0.61 (0.03)* | 0.65 (0.04)* |
| **Acting or being motivated to act to alleviate suffering** | | |
| When I'm upset, I try to do what's best for myself. | 0.86 (0.01)* | 0.83 (0.02)* |
| When I'm going through a difficult time, I try to look after myself. | 0.91 (0.01)* | 0.87 (0.02)* |
| I try to make myself feel better when I'm distressed, even if I can't do anything about the cause. | 0.76 (0.02)* | 0.80 (0.02)* |
| When I'm upset, I do my best to take care of myself. | 0.88 (0.01)* | 0.85 (0.02)* |

Standard errors are given in parentheses. * $p < .001$.

Table S7
*Correlation coefficients between total scale and subscale scores on the SOCS-O and SOCS-S using available data from 371 students (Stage 4)*

| | SOCS-O | 1 | 2 | 3 | 4 | 5 | SOCS-S | 6 | 7 | 8 | 9 | 10 |
|---|---|---|---|---|---|---|---|---|---|---|---|---|
| **Sussex-Oxford Compassion for Others Scale** (SOCS-O) | - | | | | | | | | | | | |
| 1. Recognising suffering | .72*** | - | | | | | | | | | | |
| 2. Understanding the universality of suffering | .62*** | .25*** | - | | | | | | | | | |
| 3. Feeling for the person suffering | .79*** | .47*** | .31*** | - | | | | | | | | |
| 4. Tolerating uncomfortable feelings | .77*** | .46*** | .37*** | .49*** | - | | | | | | | |
| 5. Acting or being motivated to act to alleviate suffering | .83*** | .49*** | .32*** | .69*** | .61*** | - | | | | | | |
| **Sussex-Oxford Compassion for the Self Scale** (SOCS-S) | .34*** | .14** | .44*** | .18*** | .29*** | .19*** | - | | | | | |
| 6. Recognising suffering | .32*** | .26*** | .29*** | .17** | .22*** | .23*** | .58*** | - | | | | |
| 7. Understanding the universality of suffering | .43*** | .13* | .74*** | .20*** | .29*** | .22*** | .52*** | .32*** | - | | | |
| 8. Feeling for the person suffering | .21*** | .08 | .25*** | .16** | .18** | .12* | .87*** | .34*** | .22*** | - | | |
| 9. Tolerating uncomfortable feelings | .14** | .05 | .19*** | .05 | .23*** | .01 | .79*** | .25*** | .20*** | .72*** | - | |
| 10. Acting or being motivated to act to alleviate suffering | .17** | .03 | .21*** | .10 | .18** | .13* | .86*** | .29*** | .24*** | .82*** | .67*** | - |

*$p < .05$, **$p < .01$, ***$p < .001$

## Sussex-Oxford Compassion for Others Scale (SOCS-O)

**Instructions**

Below are statements describing how you might relate to **other people**. Please indicate how true the following statements are of you using the 5-point response scale (1 = Not at all true, 2 = Rarely true, 3 = Sometimes true, 4 = Often true, 5 = Always true). For example, if you think that a statement is often true of you, circle '4'.

Note: In the below items, generic terms (e.g., 'upset', 'distress', 'suffering', struggling') are used to cover a range of unpleasant emotions, such as sadness, fear, anger, frustration, guilt, shame, etc.

Please provide an answer for each statement.

| | Not at all true | Rarely true | Sometimes true | Often true | Always true |
|---|---|---|---|---|---|
| 1. I recognise when other people are feeling distressed without them having to tell me. | 1 | 2 | 3 | 4 | 5 |
| 2. I understand that everyone experiences suffering at some point in their lives. | 1 | 2 | 3 | 4 | 5 |
| 3. When someone is going through a difficult time, I feel kindly towards them. | 1 | 2 | 3 | 4 | 5 |
| 4. When someone else is upset, I try to stay open to their feelings rather than avoid them. | 1 | 2 | 3 | 4 | 5 |
| 5. When others are struggling, I try to do things that would be helpful. | 1 | 2 | 3 | 4 | 5 |
| 6. I notice when others are feeling distressed. | 1 | 2 | 3 | 4 | 5 |
| 7. I understand that feeling upset at times is part of human nature. | 1 | 2 | 3 | 4 | 5 |
| 8. When I hear about bad things happening to other people, I feel concern for their wellbeing. | 1 | 2 | 3 | 4 | 5 |
| 9. I stay with and listen to other people when they're upset even if it's hard to bear. | 1 | 2 | 3 | 4 | 5 |
| 10. When someone is going through a difficult time, I try to look after them. | 1 | 2 | 3 | 4 | 5 |
| 11. I'm quick to notice early signs of distress in others. | 1 | 2 | 3 | 4 | 5 |
| 12. Like me, I know that other people also experience struggles in life. | 1 | 2 | 3 | 4 | 5 |

| | Not at all true | Rarely true | Sometimes true | Often true | Always true |
|---|---|---|---|---|---|
| 13. When someone is upset, I try to tune in to how they're feeling. | 1 | 2 | 3 | 4 | 5 |
| 14. I connect with the suffering of others without judging them. | 1 | 2 | 3 | 4 | 5 |
| 15. When I see someone in need, I try to do what's best for them. | 1 | 2 | 3 | 4 | 5 |
| 16. I recognise signs of suffering in others. | 1 | 2 | 3 | 4 | 5 |
| 17. I know that we can all feel upset at times when we are wronged. | 1 | 2 | 3 | 4 | 5 |
| 18. I'm sensitive to other people's distress. | 1 | 2 | 3 | 4 | 5 |
| 19. When someone else is upset, I can be there for them without feeling overwhelmed by their distress. | 1 | 2 | 3 | 4 | 5 |
| 20. When I see that someone is upset, I do my best to take care of them. | 1 | 2 | 3 | 4 | 5 |

**Scoring Information**

Users may compute both total subscale scores and a total SOCS-O score. To compute total subscale scores, sum item scores within each subscale (see below for items associated with each subscale). To compute a total SOCS-O score, sum all five total subscale scores or sum all 20 item scores.

Recognising suffering items: 1, 6, 11, 16.
Understanding the universality of suffering items: 2, 7, 12, 17.
Feeling for the person suffering items: 3, 8, 13, 18.
Tolerating uncomfortable feelings items: 4, 9, 14, 19.
Acting or being motivated to act to alleviate suffering items: 5, 10, 15, 20.

## Sussex-Oxford Compassion for the Self Scale (SOCS-S)

### Instructions

Below are statements describing how you might relate to **<u>yourself</u>**. Please indicate how true the following statements are of you using the 5-point response scale (1 = Not at all true, 2 = Rarely true, 3 = Sometimes true, 4 = Often true, 5 = Always true). For example, if you think that a statement is often true of you, circle ‘4’.

<u>Note:</u> In the below items, generic terms (e.g., ‘upset’, ‘distress’, ‘suffering’, struggling’) are used to cover a range of unpleasant emotions, such as sadness, fear, anger, frustration, guilt, shame, etc.

Please provide an answer for each statement.

| | Not at all true | Rarely true | Sometimes true | Often true | Always true |
|---|---|---|---|---|---|
| 1. I’m good at recognising when I’m feeling distressed. | 1 | 2 | 3 | 4 | 5 |
| 2. I understand that everyone experiences suffering at some point in their lives. | 1 | 2 | 3 | 4 | 5 |
| 3. When I’m going through a difficult time, I feel kindly towards myself. | 1 | 2 | 3 | 4 | 5 |
| 4. When I’m upset, I try to stay open to my feelings rather than avoid them. | 1 | 2 | 3 | 4 | 5 |
| 5. I try to make myself feel better when I’m distressed, even if I can’t do anything about the cause. | 1 | 2 | 3 | 4 | 5 |
| 6. I notice when I’m feeling distressed. | 1 | 2 | 3 | 4 | 5 |
| 7. I understand that feeling upset at times is part of human nature. | 1 | 2 | 3 | 4 | 5 |
| 8. When bad things happen to me, I feel caring towards myself. | 1 | 2 | 3 | 4 | 5 |
| 9. I connect with my own distress without letting it overwhelm me. | 1 | 2 | 3 | 4 | 5 |
| 10. When I’m going through a difficult time, I try to look after myself. | 1 | 2 | 3 | 4 | 5 |
| 11. I’m quick to notice early signs of distress in myself. | 1 | 2 | 3 | 4 | 5 |
| 12. Like me, I know that other people also experience struggles in life. | 1 | 2 | 3 | 4 | 5 |

| | Not at all true | Rarely true | Sometimes true | Often true | Always true |
|---|---|---|---|---|---|
| 13. When I'm upset, I try to tune in to how I'm feeling. | 1 | 2 | 3 | 4 | 5 |
| 14. I connect with my own suffering without judging myself. | 1 | 2 | 3 | 4 | 5 |
| 15. When I'm upset, I try to do what's best for myself. | 1 | 2 | 3 | 4 | 5 |
| 16. I recognise signs of suffering in myself. | 1 | 2 | 3 | 4 | 5 |
| 17. I know that we can all feel distressed when things don't go well in our lives. | 1 | 2 | 3 | 4 | 5 |
| 18. Even when I'm disappointed with myself, I can feel warmly towards myself when I'm in distress. | 1 | 2 | 3 | 4 | 5 |
| 19. When I'm upset, I can let the emotions be there without feeling overwhelmed. | 1 | 2 | 3 | 4 | 5 |
| 20. When I'm upset, I do my best to take care of myself. | 1 | 2 | 3 | 4 | 5 |

**Scoring Information**

Users may compute both total subscale scores and a total SOCS-S score. To compute total subscale scores, sum item scores within each subscale (see below for items associated with each subscale). To compute a total SOCS-S score, sum all five total subscale scores or sum all 20 item scores.

Recognising suffering items: 1, 6, 11, 16.
Understanding the universality of suffering items: 2, 7, 12, 17.
Feeling for the person suffering items: 3, 8, 13, 18.
Tolerating uncomfortable feelings items: 4, 9, 14, 19.
Acting or being motivated to act to alleviate suffering items: 5, 10, 15, 20.
